# Integrated Metabolomics, Lipidomics, and Genomics Reveal the Presence of a New Biomarker, Butanediol Glucuronide, Associated with the Activation of Liver Ketogenesis and Lipid Oxidation by Tomato-Based *Sofrito* in Obese Rats

**DOI:** 10.3390/antiox11112165

**Published:** 2022-10-31

**Authors:** José Fernando Rinaldi de Alvarenga, Mar Garcia-Aloy, Marynka Ulaszewska, Sebastian Zagmutt, Marta Perez-Montero, Urska Vrhovsek, Rosa M. Lamuela-Raventós, Rosalia Rodriguez-Rodriguez

**Affiliations:** 1 Human Nutrition Unit, Department of Food & Drug, University of Parma, 43124 Parma, Italy; 2 Food Research Center (FoRC), University of São Paulo, Rua do Lago 250, São Paulo 05508-080, Brazil; 3 Metabolomics Unit, Research and Innovation Centre, Fondazione Edmund Mach, 38098 San Michele all’Adige, Italy; 4 Basic Sciences Department, Faculty of Medicine and Health Sciences, Universitat Internacional de Catalunya, E-08195 Sant Cugat del Vallès, Spain; 5 Department of Nutrition, Food Science and Gastronomy, School of Pharmacy and Food Sciences, Xarxa d’Innovació Alimentària XIA, Institute of Nutrition and Food Safety (INSA-UB), University of Barcelona, 08028 Barcelona, Spain; 6 Centro de Investigación Biomédica en Red Fisiopatología de la Obesidad y la Nutrición (CIBEROBN), Instituto de Salud Carlos III, 28029 Madrid, Spain

**Keywords:** Mediterranean diet, obesity, ketogenesis, metabolomics, lipidomics, butanediol glucuronide

## Abstract

The increasing prevalence of obesity worldwide has promoted research on human metabolism and foods such as *sofrito*, a tomato and olive oil-based sauce from the Mediterranean diet, has shown beneficial effects on obesity and related complications. *Sofrito* has been associated with better cardiovascular health, metabolic syndrome, and anti-inflammatory effects. The aim of this study was to understand how *sofrito* intake could contribute to the control of energy metabolism in obese rats. For this purpose, integrative untargeted lipidomics, metabolomics, and targeted gene expression approaches were used in the liver and adipose tissue to identify metabolic changes and the mechanism of action promoted by *sofrito* intake. A new biomarker was identified in the liver, butanediol glucuronide, an indicator of ketogenic activation and lipid oxidation after the *sofrito* intervention. Gene expression analysis revealed an increase in the uptake and liver oxidation of lipids for energy production and ketogenesis activation as fuel for other tissues in *sofrito*-fed animals. *Sofrito* altered the lipidomic profile in the fat depots of obese rats. This multiomics study identifies a new biomarker linked to the beneficial actions of *sofrito* against obesity and provides further insight into the beneficial effect of the Mediterranean diet components.

## 1. Introduction

Over the last three decades, the prevalence of obesity has increased rapidly around the world [1]. In 2016, more than 1.9 billion adults, approximately 39% of the world population, were considered overweight and 650 million, about 13%, were obese [2]. Models indicate that by 2030, there will be a 33% increase in the obesity prevalence [3,4]. Obesity is established by a chronical positive caloric balance, which is associated with white adipose tissue hypertrophy and the accumulation of ectopic fat, leading to the progression of systemic inflammation, nonalcoholic fatty liver disease, and insulin resistance [5].

Up to now, the management of obesity has been based, on the one hand, on lifestyle approaches with restrictions in caloric intake and the promotion of physical activity and, on the other hand, on pharmacological and surgical interventions when the first approaches do not ameliorate obesity progression and the development of metabolic and cardiovascular complications. The clinical limitations of pharmacological and surgical treatments, including a lack of long-term therapeutic efficacy, restricted eligibility, and high economic costs [6,7,8], have positioned dietary management of obesity as an emerging approach against this prevalent disease [7]. Among these dietary-based strategies, the Mediterranean diet has shown promising effects as part of the treatment of obesity, non-alcoholic fat liver disease, and cardiovascular complications in pre-clinical and clinical trials [9,10,11]. The tomato sauce called *sofrito* is a key component of the Mediterranean diet and its consumption is one of the items to be considered when evaluating a Mediterranean diet score [12,13]. This sauce has a high content of carotenoids and phenolic compounds, and its unique method of preparation can modulate the profile of bioactive compounds and their beneficial effects [13,14,15,16]. These effects were also confirmed in in vitro studies with different cells lines for reactive oxygen species scavenging, eicosanoid production, and LDL oxidation [17,18] and also in humans, showing that a single dose of *sofrito* significantly reduces the plasmatic levels of proinflammatory biomarkers [19]. These findings are in line with the antioxidant, anti-inflammatory, and metabolic properties of tomato sauces [20,21]. Previous publications of our group have shown that chronic administration of a *sofrito*-enriched diet in obese Zucker rats is able to induce a significant improvement of vascular function and insulin sensitivity, attenuation of FGF21 resistance in white adipose tissue, and, interestingly, without changes in body weight gain despite higher caloric intake [22,23]. Thus, understanding how *sofrito* could facilitate a more favorable metabolic environment should be considered as a tool to face obesity. Particularly, although obese animals supplemented with *sofrito* (OS) showed higher caloric intake compared to the obese control group (OC) (Table S2), this hyperphagia did not imply a higher body weight gain or liver and white adipose tissue weights in relation to OC. Supplementation with *sofrito* results in the presence of bioactive compounds, such as phenolic compounds and carotenoids in feed, which were characterized in a previous publication [22] (Table S3). Therefore, to understand how this key component of the Mediterranean diet could be modulating energy metabolism in obesity, an untargeted metabolomics approach together with gene expression analysis was performed in both liver and white adipose tissue depots.

The aim of this investigation was to explore new biomarkers and study the plausible mechanism of chronic tomato-based *sofrito* intake on energy metabolism integrating metabolomics and lipidomics approaches with gene expression in obese Zucker rats. As far as we know, this is the first multiomics approach to this key food component of the Mediterranean diet.

## 2. Materials and Methods

### 2.1. Standards and Reagents

Phenolic compounds standards were purchased from Extrasynthese (Genay, France) and Sigma-Aldrich (St. Louis, MO, USA). Carotenoid standards were purchased from Sigma-Aldrich. Deuterated standards were purchased from Sigma-Aldrich and Spectra2000. Solvents were purchased from AppliChem, Panreac Quimica SA (Barcelona, Spain), Sigma-Aldrich, and Trizol Reagent for RNA extraction was supplied by Fisher Scientific (Madrid, Spain) and the SYBR® Green assay for RT-PCR analysis by Bio-Rad Laboratories (Billerica, MA, USA) and primers were provided by IDT DNA Technologies (Leuven, Belgium). Ultra-pure water was produced by a Millipore system (Millipore, Bedford, MA, USA). More details are given in the Supplementary Materials S1.

### 2.2. Animal Study

Six-week-old male obese Zucker rats and their lean littermate controls were purchased from Charles River (Charles River Laboratories, Barcelona, Spain). At 8 weeks of age, obese and lean rats were randomly assigned to the following groups (n=5): lean rats fed chow diet (LC), obese rats fed control chow diet (OC), lean rats fed chow diet supplemented in 2% (*w/w*) of *sofrito* (LS), and obese rats fed chow diet supplemented in 2% (*w/w*) of *sofrito*. Control chow diet (Teklad Global 2018) was provided by Harlan Laboratories (Milan, Italy) and *sofrito* that was used to supplement the chow diet was furnished by Gallina Blanca-Star (Barcelona, Spain). Animals were fed ad libitum. The supplementation was calculated according to the consumption of tomato by the human population, in which 2.25 g/kg of *sofrito* per week was administered [22].

Food intake and body weight were evaluated weekly. After 8 weeks of the diet intervention, animals were sacrificed by decapitation. Blood samples were collected in the moment and liver, visceral (perirenal plus retroperitoneal), and epididymal adipose tissues were dissected. All animal handling and experimentation was performed according to the European Union guidelines for the ethical management of animals and was approved by the committee of Ethical Experimentation of the Universitat de Barcelona (557/16).

### 2.3. Sofrito Bioactive Compounds Analysis in Feed

Carotenoid analyses were performed by an LC-DAD method [13] and identified by retention time chromatography with standards, UV/VIS absorption spectrum, spectral fine structure, and peak cis intensity compared to standards and the literature [14]. To confirm the identification, an HPLC-APCI-QqQ-MS/MS method was used [24]. Phenolic compounds were identified and quantified by UPLC-ESI-QqQ-MS/MS using the conditions of a validated method by Di Lecce et al. [25] for tomato polyphenols and a method described by Capriotti et al. [26]. The results were expressed as µg/g of *sofrito*. More details of the chromatographic separation and mass conditions are given in the Supplementary Materials S2.

### 2.4. Untargeted Approach

The untargeted analysis (metabolomics and lipidomics) was performed using an Orbitrap LTQ-XL (Thermo Fisher, Bremen, Germany), interfaced to a Dionex Ultimate 3000 system, consisting of an autosampler and quaternary gradient HPLC-pump. Mass measurements were acquired in centroid mode and in both positive and negative ionization modes. The samples were injected twice. The first injection was dedicated to the acquisition of full scan spectra at a resolution of 30,000 at *m/z* 400 while the second injection was dedicated to the acquisition of high-resolution MS/MS data under data-dependent acquisition (DDA) mode. In DDA mode, the resolving power for both the MS and MS2 scan was 7500 at a collision energy of CID 35eV using an isolation window of 2Da. The conditions in ESI positive (and negative) mode were source voltage 5.0 kV (3.5 kV), heated capillary temperature 320 C, capillary voltage 30 V (−30 V), and tube lens 110 V (−110 V). In the LTQ component of the instrument, nitrogen was used as both the sheath gas (70 U) and auxiliary gas (30 U), and helium was used as the damping gas. All measurements were carried out using the automatic gain control of LTQ to adjust the number of ions entering the trap.

To ensure data quality, a quality control (QC) with an equitable mixture of all different extracts was prepared and a mix of deuterated internal standards were used to fortify the samples, with IS-1 for metabolomics and IS-2 for lipidomics (Supplementary Materials S1). Quality controls were injected before, during, and once the sequence was finished to control the retention time shifts and mass accuracy. The QC injections were also used to verify the analytical variability and injection order effect.

**Figure 1:**
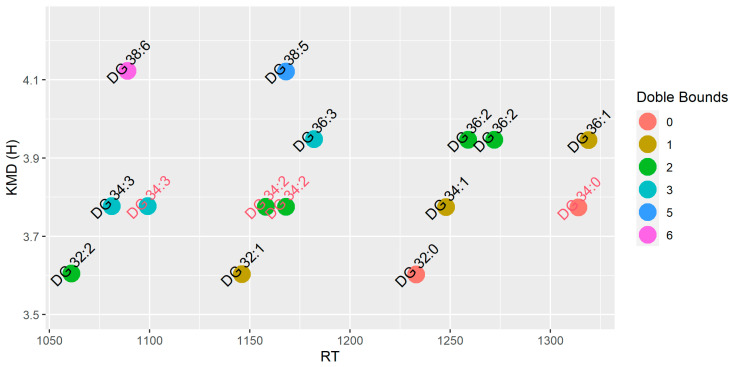
Kendricks Mass Defect calculated by the hydrogen base for diacylglycerols tentatively identified by the lipidomics approach. Metabolites were colored by doble bounds. Compounds name without MS/MS experiments were colored in red.

**Figure 2:**
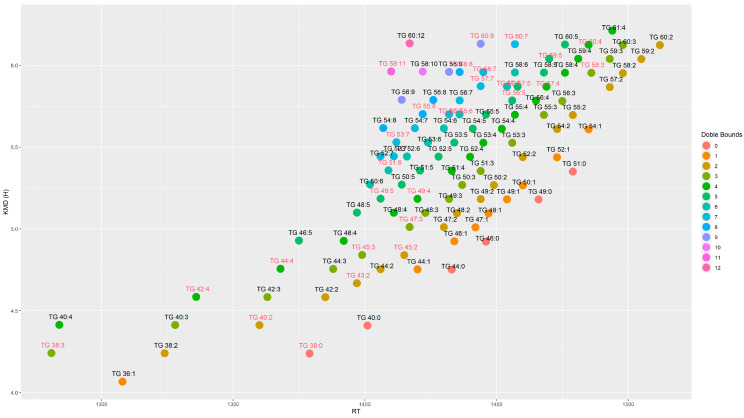
Kendricks Mass Defect calculated by the hydrogen base for triacylglycerols tentatively identified by the lipidomics approach. Metabolites were colored by doble bounds. Compound names without MS/MS experiments are colored in red.

Metabolite identifications were performed by the detected pseudo-molecular ion with a mass accuracy of 5 ppm and the isotopic pattern was checked with the theoretical isotope profile. Identification was confirmed by MS/MS experiments and comparison of the spectra with different spectral databases such as mzCloud and the literature. Isotopes and adducts were annotated for corroborate identification. Metabolites were classified according to metabolomics guidelines using four levels of identification [27]. For the lipidomics approach, the identification was further corroborated by Kendricks Mass Defect (KMD) calculated by the hydrogen base and graphs were plotted to eliminate possible misidentification (Figure 1 and Figure 2) [28]. The raw data from metabolomics and lipidomics are available at the metabolights repository MTBLS5983 (www.ebi.ac.uk/metabolights/MTBLS5983, accessed on 18 October 2022) [29].

#### 2.4.1. Metabolomics Assay

##### Sample Extraction

Plasma (50 µL) was spiked with 50 µL of IS and extracted with 150 µL of methanol:acetonitrile (1:1, *v/v*), after which it was vortexed for 10 min, with 1000 rpm at 4 °C and then centrifuged at 14,000 rpm, for 10 min at 4 °C. The supernatant was collected, and the extraction was repeated. Both supernatants were combined and evaporated until dry under a gentle nitrogen steam. The residue was reconstituted with 50 µL of external standard in methanol and 50 µL of ultrapure water was added. The extracts were transferred in amber vials with inserts and storage at −80 °C until analysis.

For liver extraction, tissue samples (70–200 mg) were weighed, frozen in nitrogen liquid, and immediately homogenized using a cryomill (Retsch®), using a frequency of 40 Hz for 10s. After that, samples were extracted using methanol:acetonitrile (1:1, *v/v*), in a proportion of solid:liquid 1 mg:5 µL, vortexed, and centrifuged in the same conditions as the plasma extraction. The extraction was performed twice and both supernatants were combined. Then, 500 µL of each extract was evaporated under nitrogen flow until dry and resuspended in 100 µL of methanol with IS, 100 µL of ultrapure water, and 50 µL of isopropanol. The extracts were transferred in amber vials with inserts and stored at −80 °C until analysis.

##### LC-HRMS Analysis

Chromatography separation was accomplished with a Kinetex C_18_ column 2.1 × 150 mm, 2.6 µm (Phenomenex). Gradient elution for metabolite separation was carried out with water 0.1% formic acid (A) and acetonitrile 0.1% formic acid (B), with a flow rate of 300 µL/min using the following gradient: 0.0 min, 95%A; 1 min, 95%A; 12.0 min, 0%A; 14.0 min, 0%A, 14.2 min, 95%A; 15.0 min, 95%A. The column temperature was maintained at 40 ºC and the injection volume was 5 µL [30]. The full scan injections were carried out within the range of 80–800 *m/z*.

#### 2.4.2. Lipidomics Assay

##### Sample Extraction

Adipose tissue, epididymal and visceral, were weighed (100~220 mg) and extracted according to the Folch method with chloroform:methanol (2:1) in 1 mg:5 µL, vortexed, and centrifuged. The lower lipid-rich layer was collected, and a second extraction was performed. Both lipid-rich layers were combined. An aliquot of 10 µL of the extract was solubilized in 150 µL of IS and 340 µL of isopropanol and analyzed [31,32].

##### LC-HRMS Analysis

Lipids separation was performed using a Kinetex C_18_ column 2.1 × 150 mm, 2.6 µm (Phenomenex), applying a gradient elution with acetonitrile: water (2:3, *v/v*) 10 mmol ammonium formate at pH =3.9 (A) and acetonitrile:isopropanol (1:9, *v/v*) 10 mmol ammonium formate at pH = 6.4 (B), with a flow rate of 200 µL/min using the follow conditions: 0.0 min, 68%A; 1.5 min, 68%A; 4.0 min, 55%A; 5.0 min, 48%A, 8.0 min, 42%A; 12.0 min, 34%A; 14.0 min, 30%A; 18 min, 25%A; 21.0 3%A; 25.0 min, 3%A; 25.1 min, 68%A, 30.0 min, 68%A. The column temperature was maintained at 55 ºC and the injection volume was 2 µL [33]. In this case, in the full scan mode, the acquisition mass range was 100 to 1000 *m/z*.

#### 2.4.3. Data Analysis

LC-MS raw files were converted to mzXML format using the MSConverter module of ProteoWizard software. FS files were further converted to mzData format to eliminate orbitrap artifacts using the functions available at https://gitlab.com/R_packages/chemhelper/blob/master/R/orbi.filter.R. Then, the mzData files were processed with the XCMS-R package [34,35,36] separately for negative and positive mode. Peak picking was performed using the “centWave” method and the following parameters were set: mass tolerance at 20 ppm, peak width range 2–40 s, prefilter range 5/5000 scans/intensity, signal-to-noise threshold 5, and noise 2000.

The processed data was first filtered by excluding features that were also present in the solvent samples by selecting those features for which the mean value within the study samples was at least twice the corresponding mean value within the solvent samples. A second filter associated with sample representativeness was applied using the 75% rule, which consisted of retaining those features that were consistently found in at least 75% of the samples of at least one experimental group. A third filter was applied using the coefficient of variation (CV), excluding those features for which their CV was higher in the QC samples than in the study samples. Statistical analysis of the data was performed by applying feature-wise multiple linear regression using the “limma” R package [37] and the *p*-values were adjusted by the Benjamini–Hochberg method to control for the false discovery rate.

### 2.5. RNA Isolation and Quantitative RT-PCR

Total RNA was extracted from liver or adipose tissues using Trizol Reagent. Retrotranscription and quantitative RT-PCR (qPCR) were performed as previously described [38]. Relative mRNA levels were measured using the CFX96 Real-Time System, C1000 Thermal Cycler (BioRad, Hercules, CA, USA). The primer sequences used are shown in Table S1. Relative gene expression was estimated using the comparative Ct (2^−ΔΔct^) method in relation to β-actin and S18 levels. The gene expression assays are expressed as the mRNA relative levels and referred to 1 assigned to lean or obese control rats, as indicated. Significant differences were assessed by a two-way ANOVA.

## 3. Results and Discussion

**Figure 3:**
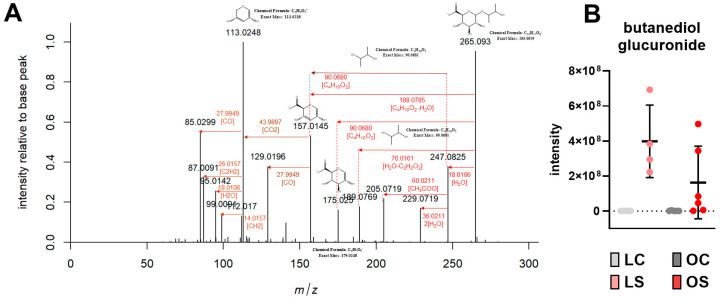
Metabolomics results in liver. Fragmentation pattern of the metabolite C010 (*m/z* 265.0930) tentatively identified as butanediol glucuronide (**A**); Intensity of the metabolite C010, as butanediol glucuronide, in the liver samples of the animals with differences in diet (*p* < 0.05) (**B**). LC, lean control; LS, lean supplemented with *sofrito*; OC, obese control; OS, obese supplemented with *sofrito*.

The untargeted analysis did not show any signals with statistically significant differences between the studied groups for plasma (data not shown). In contrast, the liver samples revealed a difference in relation to the *sofrito* consumption, but there was no difference in relation to the health status (obese and eutrophic). Most of the discriminant features corresponded to the metabolite C001 (Table 1). The C001 metabolite shown in the negative and positive mode indicated several significant signals between pseudo-molecular ion, isotopes, and adducts, with the main marker being explored. The most intense ion detected in negative mode was *m*/*z* 265.0928, corresponding to the deprotonated ion [M-H]-, and showing fragments at *m*/*z* 247.0825 and *m*/*z* 229.0719 associated with two consecutive losses of water moieties in the MS/MS spectra (Figure 3A, Table 1). The presence of a fragment at *m*/*z* 175.0250, along with fragments at *m*/*z* 113.0248, *m*/*z* 99.0091, *m*/*z* 95.0142, *m*/*z* 87.0091, and *m*/*z* 85.0299 revealed a glucuronic moiety in the molecule (Figure 3A, Table 1). Therefore, the neutral loss of 90.0680 indicates the conjugate free metabolite, with a possible molecular formula of C_4_H_10_O_2_ (Figure 1). In FS of positive ionization mode, the ion with the highest intensity corresponded to the ammonia adduct, which was found at *m/z* 284.1341. Similarly, the fragmentation spectra showed two consecutive water moiety losses. The fragment at *m/z* 91.0752 indicates the aglycone-free metabolite, with its fragment *m/z* 73.0646. This metabolite was tentatively identified as butanediol glucuronide. The plot of the peak intensities of butanediol glucuronide confirms its presence only in the animals that were supplemented with *sofrito* (LS and OS) (Figure 3B). As shown in the figure, there is an increasing trend in the LS group in comparison to the OS group, but a statistical level of significance was not reached. This compound was not detected in *sofrito* (data not shown) or its ingredients [39,40,41]. Considering this, we may speculate that butanediol glucuronide is a result of co-metabolism of the host and gut microbiome. As reviewed by Ji et al. [42], butanediol can be produced by a variety of microbiota species in the gut such as *Enterobacter* species through the anaerobic fermentation of glucose. After uptake from the intestine, it may enter the circulation and subsequently can undergo conjugation to the glucuronide moiety in the liver. Hossain et al. [43] reported a high content of butanediol as a result of pumpkin extract fermentation by *Bacillus subtilis* HA and *Lactobacillus plantarum* EJ2014. In the same study, the authors demonstrated that the administration of this butanediol-rich fermented pumpkin extract to animals fed a high-fat diet promoted a lower accumulation of fat in different depots, decreased free fatty acids, and improved the lipid profile in plasma [43]. The pumpkin extract also led to modulation of the expression of PPARγ, a key gene in the control of energy expenditure in white adipose tissue, indicating a possible bioactivity of butanediol.

Obesity development is characterized by adipose tissue hypertrophy and the accumulation of ectopic fat, which interferes with cellular and organ functions [44]. Adipose tissue hypertrophy is associated with increased inflammation, high rates of lipolysis, and insulin resistance. When reaching its maximum capacity of lipid accumulation, adipose tissue redirects lipids to other organs, especially to the liver, leading to dyslipidemia and hepatic problems [5]. A potential strategy to attenuate free fatty acids released by adipocytes is to reduce the lipolysis or to increase the ability to oxidize fatty acids by the β-oxidation process, increasing the mitochondrial content and adipose tissue browning; that is, the induction of thermogenically active adipocytes in white fat depots [5]. To explore these mechanisms, the metabolomics and lipidomics assays performed in both liver and adipose tissue were further confirmed by gene expression in both tissues.

**Figure 4:**
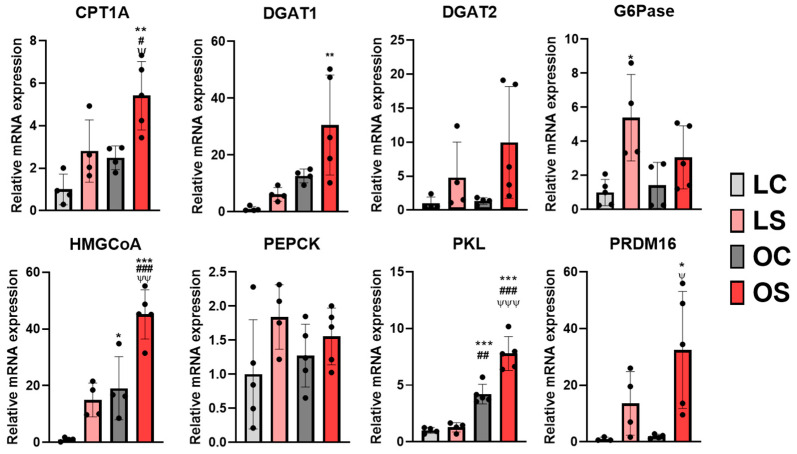
Gene expression in the liver of glucose, lipid, and ketogenic metabolism. LC, lean control; LS, lean supplemented with *sofrito*; OC, obese control; OS, obese supplemented with *sofrito*. * *p* < 0.05, ** *p* < 0.01, *** *p* < 0.001 vs. LC; # *p* < 0.05, ## *p* < 0.01, ### *p* < 0.001 vs. LS; ψ *p* < 0.05, ψψ *p* < 0.01, ψψψ *p* < 0.001 vs. OC. CPT1A, carnitine palmitoyltransferase 1A; DGAT1, diacylglycerol O-acyltransferase 1; DGAT2, diacylglycerol O-acyltransferase 2; G6Pase, glucose 6-phosphatase; HMGCoA, 3-hydroxy-3-methylglutaryl coenzyme A; PEPCK, phosphoenolpyruvate carboxykinase; PKL, piruvate kinase; PRDM16, PR domain-containing 16.

First, to understand the role of butanediol found in *sofrito*-fed rats in metabolism, gene expression analysis focused on liver energy metabolism was performed. The results indicated an increase in the expression of the esterification enzymes mediating the synthesis of TG from DG, DGAT1 and DGAT2, in lean and obese animals that were supplemented with *sofrito* in relation to their controls (Figure 4), being particularly upregulated in the OS group (Figure 4). This increment may suggest a decrease in circulating free fatty acids for triglyceride synthesis in the liver, a result that is associated with a significant increase in CPT1A and PRDM16 expression in the groups supplemented with *sofrito*, especially in obese rats, indicating a higher rate of fatty acid oxidation and higher mitochondrial function, respectively (Figure 4). In line with these results, a *sofrito*-based diet could contribute to the removal of circulating free fatty acids and their use as a source of energy. DGAT1, which is upregulated in OS, has been also described to play an important role in recycling fatty acids hydrolyzed from triglycerides in cells, protecting them from accumulation, supporting the beneficial role of *sofrito* on fatty acids metabolism and fat distribution [45].

Clinical interest has emerged in the use of therapeutic strategies able to increase liver fat oxidation, including ketogenic diets, intermittent fasting, and pharmacotherapies to treat obesity, insulin resistance, and non-alcoholic fat liver disease [46]. It has recently been reported that a decreased mitochondrial fuel supply in the liver may optimize the balance between energy supply and demand in a way that may not decrease steatosis but may decrease tissue damage and insulin resistance [46,47]. Ketogenic diets, in which carbohydrates are absent and calories restricted, generate ketone bodies, the primary source of energy for the oxidation of free fatty acids [46,48]. Recently, ketone bodies have been suggested as a fuel for mitochondria and a prominent activator of mitochondrial bioenergetics in adipose tissue that could be a tool in obesity control [49]. Butanediol has been described as a precursor molecule of β-hydroxybutyrate, a ketone body, which plays significant roles in energy homeostasis, being used as an oxidative fuel, lipogenic precursor, and signaling molecule. β-hydroxybutyrate is predominantly synthesized in the liver, being the most abundant ketone body in the circulation, and transported to other tissues for conversion into energy [46,48]. In an established obesity state, insulin resistance can lead to a lack of energy in peripheral tissues and the production of ketone bodies could be a compensatory mechanism. In our study, the ITT test performed on the experimental animals revealed a better response of the OS group animals in relation to the OC group, indicating an improvement in insulin resistance (Table S2). The increase in liver G6Pase expression in the LS group may indicate an activation of gluconeogenesis by *sofrito*, whereas this increase was not significant in the OS group in relation to OC, probably due to insulin resistance (Figure 4), and no changes were observed in PEPCK expression, another indicator of the gluconeogenic rate in the liver. On the other hand, there was a significant increase in the expression of HMGCoA and PKL in the liver of the OS group compared to the OC and lean groups, which indicates activation in the ketogenic pathway (Figure 4). These findings suggest that butanediol could be acting as a substrate by metabolism to produce hydroxybutyrate in a ketogenic process and its excess is eliminated in the form of glucuronide or is a final product of energy metabolism due to the activation of different pathways by *sofrito*.

**Figure 5:**
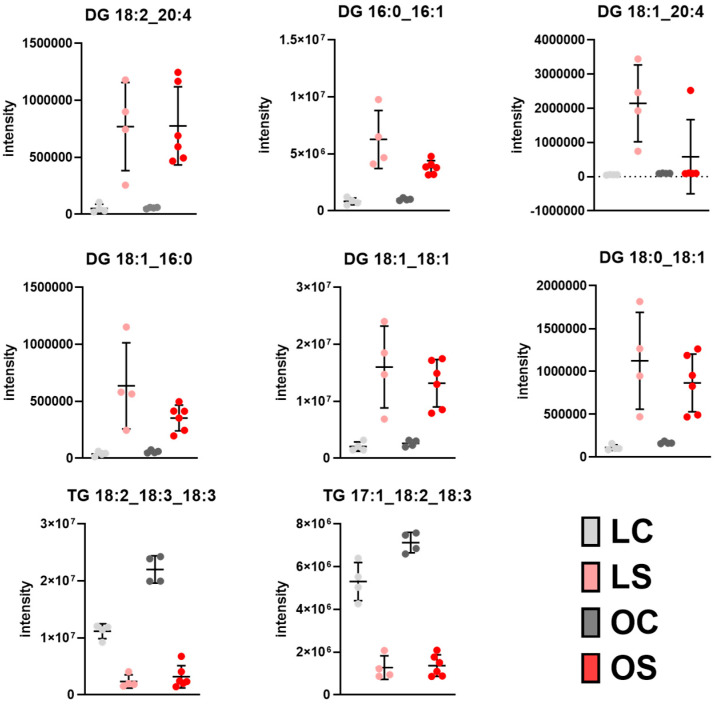
Intensity boxplot of lipids identified by the lipidomics approach in epididymal adipose tissue with differences in diet (*p* < 0.05). LC, lean control; LS, lean supplemented with sofrito; OC, obese control; OS, obese supplemented with *sofrito*.

**Figure 6:**
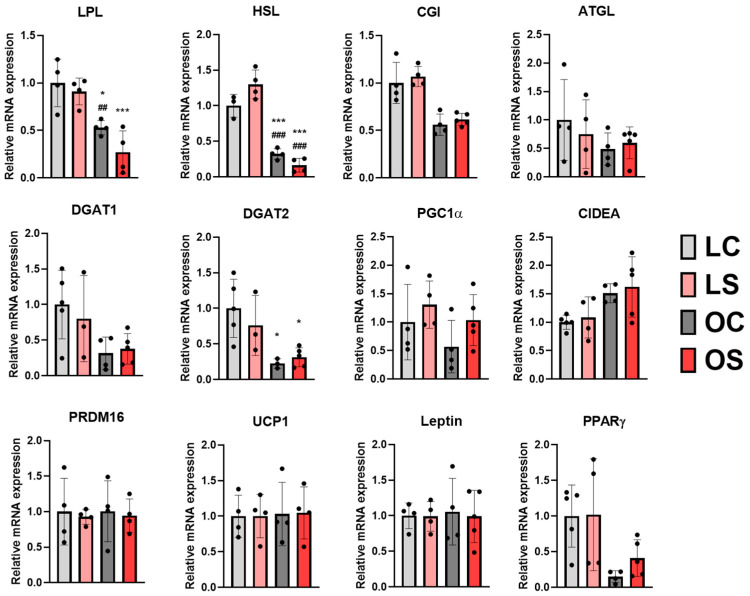
Gene expression lipid metabolism and mitochondrial function in epididymal adipose tissue. LC, lean control; LS, lean supplemented with *sofrito*; OC, obese control; OS, obese supplemented with *sofrito*. * *p* < 0.05, *** *p* < 0.001 vs. LC; ## *p* < 0.01, ### *p* < 0.001 vs. LS. ATGL, adipose triglyceride lipase; CGI, comparative gene identification 58 (α/β hydrolase); CIDEA, cell death activator; DGAT1, diacylglycerol O-acyltransferase 1; DGAT2, diacylglycerol O-acyltransferase 2; HSL, hormone-sensitive lipase; LPL, lipoprotein lipase; PRDM16, PR domain-containing 16, PGC1α, peroxisome proliferator-activated receptor gamma coactivator 1-alpha, PPARγ, peroxisome proliferator-activated receptor gamma; UCP1, uncoupling protein 1.

Activation of the ketogenic process could also act as a crosstalk between liver and adipose tissue. Then, we performed lipidomic analysis in both epididymal and visceral white adipose tissues. These results indicated statistically significant differences for 15 diglycerides (DGs) and 106 triglycerides (TGs) between both fat depots, which were annotated at the level of confidence 3, relying on the precursor ion mass accuracy, fragmentation pattern (Table 1), and relationship between the retention time and KMD(H) (Figure 1 and Figure 2). On the one hand, epididymal white adipose tissue displayed changes in its composition depending on the type of diet (control vs. *sofrito*) (Table 1, Figure 5). In general, the *sofrito*-fed rats had diglycerides as markers in the tissue composition, indicated by 12 molecules that represented 80% of the total DGs in the lipidomics, while the control diet-fed rats had triglycerides as the majority in the tissue composition, consisting of 52 different molecules that represented 52% of the identified TGs (Table 1). In epididymal adipose tissue, gene expression analysis revealed a significant decrease in the LPL and HSL mRNA levels in obese animals, which was more pronounced in the OS group (Figure 6), indicating an attenuation of the lipolysis process in OS. These two genes are related to the process of fatty acid production in a non-selective way, indicating a reduction in the lipolysis of TGs and DGs to increase storage. The lower lipolytic action is also associated with the action of ketone bodies in *sofrito*-fed rats since β-hydroxybutyrate has been described as an inhibitor of lipolysis in adipocytes by activating GRP109R, which helps to reduce circulating fatty acids [46,50]. Furthermore, the lower trend of mRNA expression levels of CGI, a co-factor ATGL, in epididymal adipose tissue found in obese animals compared to lean, although without statistical significance, could contribute to non-activation of the TGs hydrolysis process (Figure 6). The presence of a higher DG content in animals supplemented with *sofrito* by lipidomics could suggest a lower uptake of fatty acids and storage by the tissue, since both obese groups have the same expression of DGAT1 and DGAT2 but with different lipid profiles (Figure 1). This result could indicate that the modulation of the composition of epididymal white adipose tissue is not a direct consequence of the lipolysis process by the inhibition of ketone bodies and could involve other metabolic pathways in the tissue. Therefore, there is no activation of the lipolysis process nor of the synthesis or hydrolysis of TGs, suggesting a higher content of DGs in the animals supplemented with *sofrito*. In this way, the free fatty acids that reach the adipose tissue are not being used for storage but could be used by another pathway. Thus, the modulation of the lipid profile could involve the bioactive compounds present in the *sofrito*. Lipidomics also reported that control animals showed a higher TG content versus *sofrito*, with some TGs being more abundant in the OC group compared to the LC group such as TG(18:2_18:3_18:3) and TG(17:1_18:2_18:3) (Figure 5).

When analyzing the expression pattern of genes related to mitochondrial activity, the browning process, and adipocyte function (PGC1α, CIDEA, PRDM16, UCP1, leptin, and PPARγ), no significant differences were appreciated when comparing lean vs. obese and control vs. *sofrito*, despite the tendency for upregulation of these genes in the OS group compared to OC for PGC1α and PPARγ (Figure 6). These genes may indicate activation of the adipocyte energy metabolism in OS animals, stimulating glucose metabolism, mitochondrial biogenesis, and the insulin response. However, without UCP1 regulation, we can rule out the hypothesis that fatty acids can be used to generate heat by mitochondria under the *sofrito*-fed condition in epididymal white adipose tissue. The lack of browning induction in this fat depot agrees with previous investigations exploring the metabolic response of the different adipose tissues of obese mice at different temperatures, showing a lower level of thermogenic activation in epididymal depots compared to retroperitoneal depots in response to low temperatures [51,52].

**Figure 7:**
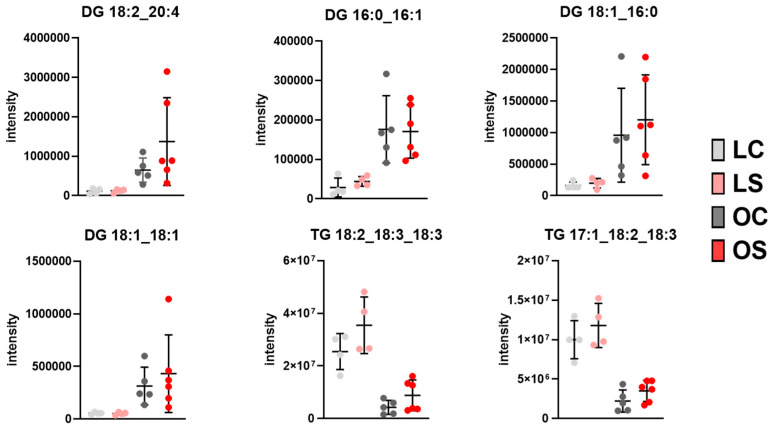
Intensity boxplot of lipids identified by the lipidomics approach in visceral adipose tissue with differences in health (*p* < 0.05). LC, lean control; LS, lean supplemented with sofrito; OC, obese control; OS, obese supplemented with *sofrito*.

On the other hand, visceral white adipose tissue showed differences in its composition in relation to the health status of the animals, regardless of the type of diet. In this fat deposit, obese animals showed DGs as markers of the tissue composition, represented by 9 DGs that make up 60% of the total identified DGs. Regarding the composition of triglycerides, a total of 20 different triglycerides were detected as markers of obese versus lean while lean groups had a higher content of 81 different TGs compared to obese groups (Table 1). It is noteworthy that the TG markers for the obese groups showed fatty acids with a small carbon chain compared to lean, with a predominance of palmitic acid (16:0) and unsaturation (16:1) (Table 1). Visceral adipose tissue is considered the main storage destination during the obesity process. The presence of higher levels of DGs (Figure 7) in obese animals may be indicative of tissue saturation in which the triglyceride production process is limited, and fatty acids can be sent to other tissues. Grzybek et al. [53] confirmed that animals fed a high-fat diet showed a higher content of diglycerides in visceral fat depots when obesity was induced compared to eutrophic animals by lipidomics analysis.

**Figure 8:**
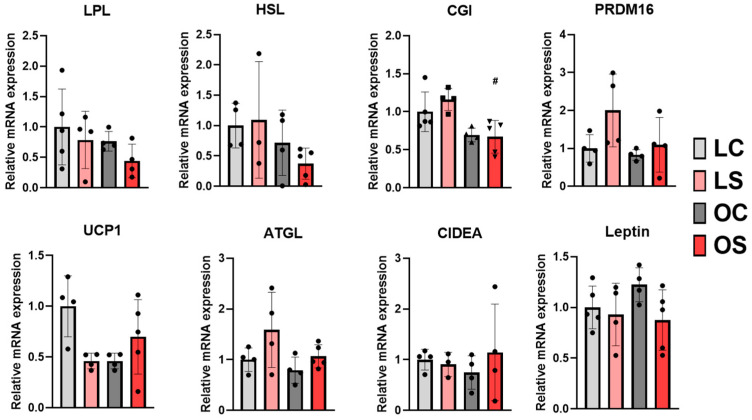
Gene expression lipid metabolism and mitochondrial function in visceral adipose tissue. LC, lean control; LS, lean supplemented with *sofrito*; OC, obese control; OS, obese supplemented with *sofrito*. # *p* < 0.05 vs. LS. ATGL, adipose triglyceride lipase; CGI, comparative gene identification 58 (α/β hydrolase); CIDEA, cell death activator; HSL, hormone-sensitive lipase; LPL, lipoprotein lipase; PRDM16, PR domain-containing 16, UCP1, uncoupling protein 1.

Gene expression analysis in this tissue revealed that there was no difference in the expression of the lipolysis proteins LPL and HSL between the groups, even with a trend of downregulation for OS compared to the others (Figure 8). When comparing the mRNA levels of the lipases, CGI revealed a significant reduction in the expression for OS rats when compared to the LS group but without significant differences in the other groups (Figure 8). This result could indicate a possible action of *sofrito* supplementation on fatty acid metabolism in fat deposits, with it being necessary to explore new pathways of action. Previous results published by Sandoval et al. [23] demonstrated a beneficial effect of *sofrito* supplementation on the resistance to FGF21 caused by obesity in visceral adipose tissue. FGF21 is an important hormone in the regulation of energy metabolism and has been pharmacologically explored for the treatment of obesity, diabetes type II, and metabolic syndrome, especially by activating the browning process [54]. In our study, we did not find significant changes in either the *sofrito* or obesity condition in the expression pattern of genes related to browning activity in visceral white adipose tissue (Figure 8). Therefore, it is not possible to associate the lipid profile of visceral adipose tissue with the induction of the browning process. Walton et al. [49] investigated the role of ketone bodies in altering the mitochondrial bioenergetics in different fat depots, showing that treatment with β-hydroxybutyrate increased the expression of genes related to thermogenesis, such as PRDM16, PGC1a, and UCP1, in adipocytes. However, the subcutaneous deposit responds better to the stimulus compared to the visceral depots, with a possible explanation being the variation in the expression of the G protein-coupled receptor.

To sum up, chronic consumption of tomato *sofrito* revealed that even with a higher caloric intake, there was no difference in the weight gain and weight of the fat deposits of the animals. This information led us to investigate the impact of this food component on energy metabolism through an untargeted metabolomics approach in the plasma and liver, revealing the metabolite butanediol glucuronide as the main biomarker after *sofrito* intake. The use of gene expression analysis indicated a modulation in the hepatic tissue, with an increase in the uptake and oxidation of lipids for energy production and the activation of the ketone bodies pathway as a possible alternative fuel for non-hepatic tissues in *sofrito*-fed animals. The lipidomics analysis revealed a difference in the epididymal white adipose tissue by the consumption of *sofrito*, verifying a decrease in the lipolysis process that could be attributed to ketone bodies but without activation of oxidative processes. On the other hand, visceral adipose tissue showed a difference between obese and eutrophic individuals, but even with tendencies to oxidative processes in animals supplemented with *sofrito*, this did not reflect a different tissue lipid profile. *Sofrito* intake could be used as an activator in the hepatic ketogenic process for energy homeostasis and in the control of body weight gain. The crosstalk between tissues should be further investigated to better understand the role of butanediol in weight gain regulation and fat deposit accumulation in obesity.

## 4. Conclusions

This untargeted approach revealed the presence of butanediol glucuronide as a marker for tomato *sofrito* intake in lean and obese supplemented animals. This molecule was related with activation of the ketogenic process in liver, which was confirmed by targeted gene expression by overexpression of HMGCoA and PKL in the *sofrito*-supplemented groups. The increase in the expression of CPT1A and PRDM16 in the liver of obese animals supplemented with *sofrito* also indicated fatty acids metabolism activation. The lipidomics approach was able to identify differences in the composition of epididymal adipose tissue by diet with an inhibition of the lipolysis process that could be related to the activation of ketogenesis by crosstalk between the liver and fat depots.

The use of untargeted omics approaches showed the possibility that a new biomarker identified after the consumption of *sofrito* could be contributing to its beneficial effects on obesity. The role of bioactive food compounds in the activation of energy metabolism and the browning process is already described in the literature; however, new compounds originating from microbiota and metabolism must also be elucidated. The presence of butanediol glucuronide, a precursor of ketone bodies, has been shown to activate ketogenesis in the liver and act as a mediator of crosstalk with adipose tissue, helping to understand the role of *sofrito* in the regulation of energy metabolism in obese Zucker rats. Our study indicates the potential contribution of butanediol glucuronide to monitor the response to nutritional interventions such as those with tomato-based *sofrito*, and it also suggests ketogenesis and its metabolites as a target pathway to manage obesity and related diseases. These findings provide further insight into the beneficial effect of crucial components of the Mediterranean diet in the management of metabolic diseases such as obesity.

## Figures and Tables

**Table 1:** Identification of markers in the metabolomics and lipidomics approach.

C	Compound	rt	P	MF	Exact Mass	MS/MS	Error	ID	T	Change
C001	butanediol glucuronide (alcohol)	81	−	C_10_H_18_O_8_	265.0928 [M − H]^−^ 531.1929 [2M − H]^−^ 363.0697 [M − H+H_3_PO_4_]^−^	265.0930 [M − H] (90); 247.0825 [M − H]-H_2_O; (40) 229.0719 [M − H]-(2)H_2_O (20); 205.0719 [M − H]-CH_3_COO (25); 189.0769 [M − H]-C_2_H_2_O_2_-H_2_O (20); 175.0250 [M − H]-glucuronide (20); 157.0145 [M − H]-glucuronide-H_2_O (50); 129.0196 (40); 113.0248 (100) gluruconide frag; 99.0091 (15) gluruconide frag; 95.0142 (30) gluruconide frag; 87.0091 (40) gluruconide frag; 85.0299 (60) gluruconide frag	0.30	II	L	S > C
			+		284.1341 [M + NH_4_]^+^ 267.1077 [M + H]^+^ 533.2075 [2M + H]^+^ 289.0895 [M + Na]^+^ 305.0635 [M + K]^+^	249.0965 (100) [M + H]-NH_3_-H_2_O; 91.0751 (15) [M + H]-glucuronide; 73.0645 (30); [M + H]- H_2_O-glucuronide	−1.12			
C002	DG 14:0_18:2	1061	+	C_35_H_64_O_5_	582.5090 [M + NH_4_]^+^	565.3 [M + H]-NH_3_ (25); 547.4 [M + H]-NH_3_-H_2_O (100); 337.3 (228) [M + H]-C_14_H_28_O_2_ (60); 285.2 (280.1) [M + H]-C_18_H_32_O_2_ (90)	−0.20	II	V	O > L
C003	DG 16:1_18:2	1081	+	C_37_H_66_O_5_	608.5230 [M + NH_4_]^+^ 629.4522 [M + K]^+^	591.5 [M + H]-NH_3_ (60); 573.4 [M + H]-NH_3_-H_2_O (100); 337.3 [M + H]-C_16_H_30_O_2_ (30); 311.3 [M + H]-C_18_H_32_O_2_ (20)	−2.99	II	E	S > C
C004	DG 18:2_20:4	1089	+	C_41_H_68_O_5_	658.5385 [M + NH_4_]^+^	641.5 [M + H]-NH_3_ (100); 623.4 [M + H]-NH_3_-H_2_O (25); 361.3 [M + H]-C_18_H_32_O_2_ (10); 337.3 [M + H]-C_20_H_32_O_2_ (100)	−2.99	II	E	S > C
C005	DG 34:3 (II)	1099	+	C_37_H_66_O_5_	608.5233 [M + NH_4_]^+^	n.d.	−2.48	III	E	S > C
C006	DG 16:0_16:1	1146	+	C_35_H_66_O_5_	584.5230 [M + NH_4_]^+^ 605.4523 [M + K]^+^ 612.5542 [M + C_2_H_8_N]^+^	567.4 [M + H]-NH_3_ (100); 549.5 [M + H]-NH_3_-H_2_O (95); 313.3 [M + H]-NH_3_-C_16_H_30_O_2_ (80); 311.3 [M + H]-NH_3_-C_16_H_32_O_2_ (60)	−3.12	II	V	S > C O > L
C007	DG 34:2 (I)	1158	+	C_37_H_68_O_5_	610.5385 [M + NH_4_]^+^ 615.4949 [M + Na]^+^ 638.5699 [M + C_2_H_8_N]^+^ 631.4679 [M + K]^+^	n.d.	−3.23	III	E,V	S > C
C008	DG 34:2 (II)	1168	+	C_37_H_68_O_5_	610.5384 [M + NH_4_]^+^ 615.4942 [M + Na]^+^ 631.4675 [M + K]^+^ 638.5697 [M + C_2_H_8_N]^+^	n.d.	−3.40	III	E,V	S > C
C009	DG 18:1_20:4	1168	+	C_41_H_70_O_5_	660.5542 [M + NH_4_]^+^ 681.4834 [M + K]^+^	643.4 [M + H]-NH_3_ (70); 625.5 [M + H]-NH_3_-H_2_O (20); 361.4 [M + H]-NH_3_-C_18_H_34_O_2_ (20); 339.3 [M + H]-NH_3_-C_20_H_32_O_2_ (100)	−2.90	II	E	S > C O > L
C010	DG 18:1_18:2	1182	+	C_39_H_70_O_5_	636.5540 [M + NH_4_]^+^ 641.5097 [M + Na]^+^ 664.5857 [M + C_2_H_8_N]^+^ 657.4833 [M + K]^+^	619.5 [M + H]-NH_3_ (70); 601.5 [M + H]-NH_3_-H_2_O (100); 339.2 [M + H]-NH_3_-C_18_H_32_O_2_ (35); 337.2 [M + H]-NH_3_-C_18_H_34_O_2_ (20);	−3.34	II	E	S > C
C011	DG 16:0_16:0	1233	+	C_35_H_68_O_5_	586.5387 [M + NH_4_] 614.5700 [M + C_2_H_8_N] 607.4678 [M + K]	569.5 [M + H]-NH_3_ (60); 551.5 [M + H]-NH_3_-H_2_O (70); 313.2 [M + H]-NH_3_-C_16_H_32_O_2_ (100)	−3.02	II	E,V	S > C O > L
C012	DG 16:0_18:1	1248	+	C_37_H_70_O_5_	612.5542 [M + NH_4_]^+^ 617.5096 [M + Na]^+^ 640.5854 [M + C_2_H_8_N]^+^ 633.4830 [M + K]^+^	595.3 [M + H]-NH_3_ (50); 577.4 [M + H]-NH_3_-H_2_O (100); 339.3 [M + H]-NH_3_-C_16_H_32_O_2_ (70); 313.2 [M + H]-NH_3_-C_18_H_34_O_2_ (80)	−3.14	II	E,V	S > C O > L
C013	DG 18:1_18:1	1259	+	C_39_H_72_O_5_	638.5716 [M + NH_4_]^+^ 643.5273 [M + Na]^+^ 659.5008 [M + K]^+^ 666.6033 [M + C_2_H_8_N]^+^	621.5 [M + H]-NH_3_ (35); 603.5 [M + H]-NH_3_-H_2_O (75); 339.2 [M + H]-NH_3_-C_18_H_34_O_2_ (100)	−0.19	II	E,V	S > C O > L
C014	DG 18:0_18:2	1272	+	C_39_H_72_O_5_	638.5719 [M + NH_4_]^+^ 666.6035 [M + C_2_H_8_N]^+^ 659.5008 [M + K]^+^	621.5 [M + H]-NH_3_ (20); 603.5 [M + H]-NH_3_-H_2_O (100); 341.2 [M + H]-NH_3_-C_18_H_32_O_2_ (85) 337.4 [M + H]-NH_3_-C_18_H_36_O_2_ (40)	0.30	II	V	O > L
C015	TG 38:3	1281	+	C_41_H_72_O_6_	678.5672 [M + NH_4_]^+^ 699.4965 [M + K]^+^	n.d.	0.85	III	E,V	C > S L > O
C016	TG 4:0_18:2_18:2	1284	+	C_43_H_74_O_6_	704.5814 [M + NH_4_]^+^ 7255121 [M + K]^+^ 732.6140 [M + C_2_H_8_N]^+^	687.5 [M + H]-NH_3_ (100); 669.7 [M + H]-NH_3_-H_2_O (15); 599.5 [M + H]-NH_3_-C_4_H_8_O_2_ (70) 407.4 [M + H]-NH_3_-C_18_H_32_O_2_ (65)	−1.22	II	E,V	C > S L > O
C017	TG 2:0_16:0_18:1	1308	+	C_39_H_72_O_6_	654.5647 [M + NH_4_]^+^ 675.4943 [M + K]^+^ 682.5963 [M + C_2_H_8_N]^+^	637.4 [M + H]-NH_3_ (10); 577.4 [M + H]-NH_3_-C_2_H_4_O_2_ (50) 381.3 [M + H]-NH_3_-C_16_H_32_O_2_ (100) 355.2 [M + H]-NH_3_-C_18_H_32_O_2_ (45)	−3.05	II	E	S > C
C018	DG 34:0	1314	+	C_37_H_72_O_5_	614.5716 [M + NH_4_]^+^ 635.5011 [M + K]^+^	n.d.	−0.19	III	V	O > L
C019	DG 18:0_18:1	1319	+	C_39_H_74_O_5_	640.5874 [M + NH_4_]^+^ 661.5168 [M + K]^+^ 668.6190 [M + C_2_H_8_N]^+^	623.2 [M + H]-NH_3_ (65); 605.6 [M + H]-NH_3_-H_2_O (85); 341.2 [M + H]-NH_3_-C_18_H_34_O_2_ (100); 339.3 [M + H]-NH_3_-C_18_H_36_O_2_ (70)	0.06	II	E,V	S > C O > L
C020	TG 4:0_16:0_18:2	1324	+	C_41_H_74_O_6_	680.5820 [M + NH_4_]^+^ 685.5379 [M + Na]^+^ 701.5116 [M + K]^+^ 708.6138 [M + C_2_H_8_N]^+^	575.5 [M + H]-NH_3_-C_4_H_8_O_2_ (90) 407.3 [M + H]-NH_3_-C_16_H_32_O_2_ (100) 383.4 [M + H]-NH_3_-C_18_H_32_O_2_ (70)	−0.36	II	V	L > O
C021	TG 4:0_18:1_18:2	1328	+	C_43_H_76_O_6_	706.5981 [M + NH_4_]^+^ 727.5277 [M + K]^+^ 734.6296 [M + C_2_H_8_N]^+^	689.5 [M + H]-NH_3_ (50); 671.5 [M + H]-NH_3_-H_2_O (15); 601.5 [M + H]-NH_3_-C_4_H_8_O_2_ (100) 407.3 [M + H]-NH_3_-C_18_H_32_O_2_ (50) 409.3 [M + H]-NH_3_-C_18_H_34_O_2_ (60)	0.23	II	E,V	C > S L > O
C022	TG 42:4	1336	+	C_45_H_78_O_6_	732.6140 [M + NH_4_]^+^	n.d.	0.59	III	V	L > O
C023	TG 40:2	1360	+	C_43_H_78_O_6_	708.6137 [M + NH_4_]^+^ 736.6456 [M + C_2_H_8_N]^+^	n.d.	0.17	III	V	L > O
C024	TG 6:0_18:1_18:2	1363	+	C_45_H_80_O_6_	734.6295 [M + NH_4_]^+^ 755.5590 [M + K]^+^	717.5 [M + H]-NH_3_ (40); 601.5 [M + H]-NH_3_-C_6_H_12_O_2_ (100) 437.4 [M + H]-NH_3_-C_18_H_32_O_2_ (75) 435.4 [M + H]-NH_3_-C_18_H_34_O_2_ (55)	0.38	II	E,V	C > S L > O
C025	TG 44:4	1368	+	C_47_H_82_O_6_	760.6453 [M + NH_4_]^+^ 781.5746 [M + K]^+^	n.d.	0.57	III	E,V	C > S L > O
C026	TG 10:0_18:2_18:3	1375	+	C_49_H_84_O_6_	786.6609 [M + NH_4_]^+^	769.5 [M + H]-NH_3_ (100); 751.5 [M + H]-NH_3_-H_2_O (20); 597.4 [M + H]-NH_3_-C_10_H_20_O_2_ (50) 491.5 [M + H]-NH_3_-C_18_H_30_O_2_ (35) 489.4 [M + H]-NH_3_-C_18_H_32_O_2_ (45)	0.47	II	V	L > O
C027	TG 38:0	1379	+	C_41_H_78_O_6_	684.6140 [M + NH_4_]^+^ 705.5435 [M + K]^+^	n.d.	0.63	III	E,V	C > S L > O
C028	TG 8:0_16:0_18:2	1385	+	C_45_H_82_O_6_	736.6451 [M + NH_4_]^+^ 757.5747 [M + K]^+^ 741.6006 [M + Na]^+^	719.6 [M + H]-NH_3_ (20); 575.5 [M + H]-NH_3_-C_8_H_16_O_2_ (100); 463.3 [M + H]-NH_3_-C_16_H_32_O_2_ (85) 439.3 [M + H]-NH_3_-C_18_H_32_O_2_ (75)	0.31	II	E,V	C > S L > O
C029	TG 10:0_16:1_18:2	1388	+	C_47_H_84_O_6_	762.6611 [M + NH_4_]^+^ 783.5906 [M + K]^+^	745.6 [M + H]-NH_3_ (20); 573.5 [M + H]-NH_3_-C_10_H_20_O_2_ (100) 491.4 [M + H]-NH_3_-C_16_H_30_O_2_ (80) 465.4 [M + H]-NH_3_-C_18_H_32_O_2_ (70)	0.77	II	E,V	C > S L > O
C030	TG 10:0_18:2_18:2	1392	+	C_49_H_86_O_6_	788.6765 [M + NH_4_]^+^ 809.6062 [M + K]^+^ 793.6322 [M + Na]^+^ 816.7082 [M + C_2_H_8_N]^+^	771.6 [M + H]-NH_3_ (100); 753.6 [M + H]-NH_3_-H_2_O (20); 699.5 [M + H]-NH_3_-C_10_H_20_O_2_ (90) 491.5 [M + H]-NH_3_-C_18_H_32_O_2_ (80)	0.42	II	E,V	C > S L > O
C031	TG 43:2	1397	+	C_46_H_84_O_6_	750.6611 [M + NH_4_]^+^	n.d.	0.78	III	V	L > O
C032	TG 12:0_18:2_18:3	1397	+	C_51_H_88_O_6_	814.6926 [M + NH_4_]^+^ 835.6221 [M + K]^+^	797.6 [M + H]-NH_3_ (100); 779.6 [M + H]-NH_3_-H_2_O (20); 597.5 [M + H]-NH_3_-C_12_H_24_O_2_ (50) 519.4 [M + H]-NH_3_-C_18_H_30_O_2_ (40) 517.3 [M + H]-NH_3_-C_18_H_32_O_2_ (35)	0.97	II	E,V	C > S L > O
C033	TG 45:3	1399	+	C_48_H_86_O_6_	776.6767 [M + NH_4_]^+^	n.d.	0.69	III	V	L > O
C034	TG 8:0_16:0_16:0	1401	+	C_43_H_82_O_6_	712.6453 [M + NH_4_]^+^ 733.5748 [M + K]^+^	551.5 [M + H]-NH_3_-C_8_H_16_O_2_ (50) 439.4 [M + H]-NH_3_-C_16_H_32_O_2_ (100)	0.60	II	E,V	C > S L > O
C035	TG 14:1_18:2_18:3	1402	+	C_53_H_90_O_6_	840.7079 [M + NH_4_]^+^ 861.6371 [M + K]^+^	823.6 [M + H]-NH_3_ (100); 805.5 [M + H]-NH_3_-H_2_O (20); 597.4 [M + H]-NH_3_-C_14_H_26_O_2_ (70) 545.5 [M + H]-NH_3_-C_18_H_30_O_2_ (50) 543.4 [M + H]-NH_3_-C_18_H_32_O_2_ (40)	0.51	II	E,V	C > S L > O
C036	TG 10:0_16:0_18:2	1406	+	C_47_H_86_O_6_	764.6761 [M + NH_4_]^+^ 785.6061 [M + K]^+^	747.5 [M + H]-NH_3_ (10) 575.4 [M + H]-NH_3_-C_10_H_20_O_2_ (100) 491.4 [M + H]-NH_3_-C_16_H_32_O_2_ (90) 467.5 [M + H]-NH_3_-C_18_H_32_O_2_ (80)	−0.11	II	V	L > O
C037	TG 49:5	1406	+	C_52_H_90_O_6_	828.7085 [M + NH_4_]^+^	n.d.	1.26	III	V	L > O
C038	TG 16:1_18:3_18:3	1406	+	C_55_H_92_O_6_	866.7238 [M + NH_4_]^+^ 887.6536 [M + K]^+^	849.7 [M + H]-NH_3_ (100); 831.6 [M + H]-NH_3_-H_2_O (20); 595.6 [M + H]-NH_3_-C_16_H_30_O_2_ (50) 571.4 [M + H]-NH_3_-C_18_H_30_O_2_ (60)	0.79	II	E,V	C > S L > O
C039	TG 18:2_18:3_18:3	1407	+	C_57_H_94_O_6_	892.7394 [M + NH_4_]^+^ 897.6951 [M + Na]^+^ 913.6689 [M + K]^+^ 920.7722 [M + C_2_H_8_N]^+^	875.7 [M + H]-NH_3_ (100); 857.6 [M + H]-NH_3_-H_2_O (20); 597.4 [M + H]-NH_3_-C_18_H_30_O_2_ (70); 595.4 [M + H]-NH_3_-C_18_H_32_O_2_ (40)	0.71	II	E,V	C > S L > O
C040	TG 51:6	1409	+	C_54_H_92_O_6_	854.7236 [M + NH_4_]^+^	n.d.	0.56	III	E,V	C > S L > O
C041	TG 58:11	1410	+	C_61_H_96_O_6_	942.7511 [M + NH_4_]^+^	n.d.	−3.60	III	E,V	C > S
C042	TG 14:1_16:1_18:2	1411	+	C_51_H_90_O_6_	816.7072 [M + NH_4_]^+^ 821.6636 [M + Na]^+^ 837.6373 [M + K]^+^	799.8 [M + H]-NH_3_ (80); 781.8 [M + H]-NH_3_-H_2_O (10); 573.5 [M + H]-NH_3_-C_14_H_26_O_2_ (100) 545.4 [M + H]-NH_3_-C_16_H_30_O_2_ (60) 519.5 [M + H]-NH_3_-C_18_H_32_O_2_ (50)	-0.35	II	V	L > O
C043	TG 16:0_16:3_20:4/TG 16:3_18:2_18:2	1411	+	C_55_H_92_O_6_	866.7238 [M + NH_4_]^+^ 887.6537 [M + K]^+^	849.7 [M + H]-NH_3_ (70); 831.6 [M + H]-NH_3_-H_2_O (15); 599.5 [M + H]-NH_3_-C_16_H_26_O_2_ (100) 569.3 [M + H]-NH_3_-C_18_H_32_O_2_ (40) 547.4 [M + H]-NH_3_-C_20_H_32_O_2_ (50)	0.79	III	V	L > O
C044	TG 53:7	1412	+	C_56_H_94_O_6_	880.7392 [M + NH_4_]^+^	n.d.	−0.49	III	E,V	C > S L > O
C045	TG 16:1_16:1_18:3/ TG 14:1_18:2_18:2	1414	+	C_53_H_92_O_6_	842.7227 [M + NH_4_]^+^ 847.6791 [M + Na]^+^ 863.6530 [M + K]^+^	825.7 [M + H]-NH_3_ (100); 807.7 [M + H]-NH_3_-H_2_O (20); 599.5 [M + H]-NH_3_-C_14_H_26_O_2_ (40) 571.4 [M + H]-NH_3_-C_16_H_30_O_2_ (60) 547.5 [M + H]-NH_3_-C_18_H_30_O_2_ (30) 545.4 [M + H]-NH_3_-C_18_H_32_O_2_ (50)	−0.52	II	E,V	C > S L > O
C046	TG 18:2_18:3_20:4/TG 18:2_18:2_20:5	1414	+	C_59_H_96_O_6_	918.7550 [M + NH_4_]^+^ 939.6845 [M + K]^+^	901.7 [M + H]-NH_3_ (80) 883.7 [M + H]-NH_3_-H_2_O (20) 623.5 [M + H]-NH_3_-C_18_H_30_O_2_ (20) 621.6 [M + H]-NH_3_-C_18_H_32_O_2_ (30) 599.5 [M + H]-NH_3_-C_20_H_32_O_2_ (40) 597.4 [M + H]-NH_3_-C_20_H_32_O_2_ (100)	0.63	II	E,V	C > S L > O
C047	TG 45:2	1415	+	C_48_H_88_O_6_	778.6926 [M + NH_4_]^+^	n.d.	1.01	III	V	L > O
C048	TG 16:1_18:2_18:3	1416	+	C_55_H_94_O_6_	868.7355 [M + NH_4_]^+^ 873.6923 [M + Na]^+^ 889.6655 [M + K]^+^	851.6 [M + H]-NH_3_ (100) 833.6 [M + H]-NH_3_-H_2_O (20) 597.5 [M + H]-NH_3_-C_16_H_30_O_2_ (60) 573.5 [M + H]-NH_3_-C_18_H_30_O_2_ (40) 571.5 [M + H]-NH_3_-C_18_H_32_O_2_ (35)	−0.56	II	E,V	C > S L > O
C049	TG 47:3	1417	+	C_50_H_90_O_6_	804.7088 [M + NH_4_]^+^	n.d.	1.68	III	V	L > O
C050	TG 18:2_20:4_22:6	1417	+	C_63_H_98_O_6_	968.7675 [M + NH_4_]^+^	651.6 [M + H]-NH_3_ (45) 671.5 [M + H]-NH_3_-C_18_H_32_O_2_ (30) 647.4 [M + H]-NH_3_-C_20_H_32_O_2_ (100) 623.4 [M + H]-NH_3_-C_22_H_32_O_2_ (60)	−2.71	II	E	C > S
C051	TG 18:2_18:2_18:3	1419	+	C_57_H_96_O_6_	894.7538 [M + NH_4_]^+^ 899.7105 [M + Na]^+^ 915.6837 [M + K]^+^ 922.788 [M + C_2_H_8_N]^+^	877.8 [M + H]-NH_3_ (100) 859.8 [M + H]-NH_3_-H_2_O (15) 599.4 [M + H]-NH_3_-C_18_H_30_O_2_ (70) 597.4 [M + H]-NH_3_-C_18_H_32_O_2_ (70)	−0.72	II	E,V	C > S L > O
C052	TG 10:0_16:0_18:1/ others	1420	+	C_47_H_88_O_6_	766.6919 [M + NH_4_]^+^ 771.6481 [M + Na]^+^ 787.6217 [M + K]^+^	577.4 [M + H]-NH_3_-C_10_H_20_O_2_ (50) 549.5 [M + H]-NH_3_-C_12_H_24_O_2_ (70) 521.4 [M + H]-NH_3_-C_14_H_28_O_2_ (85) 495.4 [M + H]-NH_3_-C_16_H_30_O_2_ (60) 493.4 [M + H]-NH_3_-C_16_H_32_O_2_ (100) 467.5 [M + H]-NH_3_-C_18_H_34_O_2_ (40)	0.09		E,V	S > C O > L
C053	TG 49:4	1420	+	C_52_H_92_O_6_	851.6532 [M + K]^+^ 830.7241 [M + NH_4_]^+^	n.d.	1.19	III	E,V	C > S L > O
C054	TG 15:1_18:2_18:2	1421	+	C_54_H_94_O_6_	856.7396 [M + NH_4_]^+^ 877.6690 [M + K]^+^	839.6 [M + H]-NH_3_ (100) 821.6 [M + H]-NH_3_-H_2_O (15) 599.5 [M + H]-NH_3_-C_15_H_28_O_2_ (40) 559.5 [M + H]-NH_3_-C_18_H_32_O_2_ (60)	0.98	II	E,V	C > S L > O
C055	TG 55:8	1422	+	C_58_H_96_O_6_	906.7541 [M + NH_4_]^+^	n.d.	−0.37	III	V	L > O
C056	TG 18:2_18:2_22:6	1422	+	C_61_H_98_O_6_	944.7701 [M + NH_4_]^+^ 965.6994 [M + K]^+^	927.7 [M + H]-NH_3_ (60) 909.6 [M + H]-NH_3_-H_2_O (30) 647.5 [M + H]-NH_3_-C_18_H_32_O_2_ (20) 599.5 [M + H]-NH_3_-C_22_H_32_O_2_ (100)	0.02	II	E,V	C > S L > O
C057	TG 16:1_16:1_16:1	1423	+	C_51_H_92_O_6_	818.7227 [M + NH_4_]^+^ 839.6530 [M + K]^+^ 823.6790 [M + Na]^+^	801.6 [M + H]-NH_3_ (20) 783.5 [M + H]-NH_3_-H_2_O (10) 547.4 [M + H]-NH_3_-C_16_H_30_O_2_ (100)	−0.54	II	E,V	S > C O > L
C058	TG 17:1_18:2_18:3	1424	+	C_56_H_96_O_6_	882.7549 [M + NH_4_]^+^ 903.6841 [M + K]^+^	865.7 [M + H]-NH_3_ (100); 847.6 [M + H]-NH_3_-H_2_O (20); 585.3 [M + H]-NH_3_-C_18_H_32_O_2_ (60); 597.5 [M + H]-NH_3_-C_17_H_32_O_2_ (50);	0.54	II	E,V	C > S L > O
C059	TG 18:2_18:2_20:4	1426	+	C_59_H_98_O_6_	920.7697 [M + NH_4_]^+^ 941.6994 [M + K]^+^	903.7 [M + H]-NH_3_ (65); 623.6 [M + H]-NH_3_-C_18_H_32_O_2_ (20); 599.5 [M + H]-NH_3_-C_20_H_32_O_2_ (20);	−0.42	II	E,V	C > S L > O
C060	TG 16:0_16:1_20:4	1428	+	C_55_H_96_O_6_	870.7535 [M + NH_4_]^+^ 853.7282 [M + H]^+^	853.8 [M + H]-NH_3_ (90); 835.8 [M + H]-NH_3_-H_2_O (20) 597.5 [M + H]-NH_3_-C_16_H_32_O_2_ (60) 549.5 [M + H]-NH_3_-C_20_H_32_O_2_ (100)	−1.09	II	V	L > O
C061	TG 15:0_16:1_16:1	1430	+	C_50_H_92_O_6_	806.7238 [M + NH_4_]^+^	789.3 [M + H]-NH_3_ (30); 547.4 [M + H]-NH_3_-C_15_H_30_O_2_ (30) 535.5 [M + H]-NH_3_-C_16_H_30_O_2_ (100)	0.85	III	V	O > L
C062	TG 18:2_18:2_18:2	1430	+	C_57_H_98_O_6_	896.7688 [M + NH_4_]^+^ 917.6988 [M + K]^+^ 879.7437 [M + H]^+^ 901.7257 [M + Na]^+^	879.7 [M + H]-NH_3_ (100); 861.7 [M + H]-NH_3_-H_2_O (15); 599.5 [M + H]-NH_3_-C_18_H_32_O_2_ (100);	−1.46	II	E,V	C > S L > O
C063	TG 15:0_16:1_18:2	1432	+	C_52_H_94_O_6_	832.7392 [M + NH_4_]^+^	815.7 [M + H]-NH_3_ (60); 573.5 [M + H]-NH_3_-C_15_H_30_O_2_ (100) 561.4 [M + H]-NH_3_-C_16_H_30_O_2_ (80) 535.4 [M + H]-NH_3_-C_18_H_32_O_2_ (95);	0.52	II	E,V	C > S L > O
C064	TG 55:7	1432	+	C_58_H_98_O_6_	908.7698 [M + NH_4_]^+^	n.d.	−0.31	III	E,V	C > S L > O
C065	TG 18:2_18:2_22:5/ TG 18:1_18:2_20:6	1432	+	C_61_H_100_O_6_	946.7825 [M + NH_4_]^+^	929.7 [M + H]-NH_3_ (45); 911.6 [M + H]-NH_3_-H_2_O (15); 649.6 [M + H]-NH_3_-C_18_H_32_O_2_ (40); 625.7 [M + H]-NH_3_-C_20_H_28_O_2_ (30); 599.5 [M + H]-NH_3_-C_22_H_34_O_2_ (100);	−3.48	II	E	C > S
C066	TG 12:0_14:0_18:0/ TG 14:0_14:0_16:0	1433	+	C_47_H_90_O_6_	768.7081 [M + NH_4_]^+^	551.5 [M + H]-NH_3_-C_12_H_24_O_2_ (50); 523.5 [M + H]-NH_3_-C_14_H_28_O_2_ (80); 495.5 [M + H]-NH_3_-C_16_H_32_O_2_ (100);	0.83	II	E,V	S > C O > L
C067	TG 16:1_17:1_18:2	1433	+	C_54_H_96_O_6_	858.7547 [M + NH_4_]^+^ 879.6843 [M + K]^+^	841.7 [M + H]-NH_3_ (100); 587.5 [M + H]-NH_3_-C_16_H_30_O_2_ (65); 573.4 [M + H]-NH_3_-C_17_H_32_O_2_ (75); 561.5 [M + H]-NH_3_-C_18_H_32_O_2_ (60);	0.32	II	E,V	C > S L > O
C068	TG 14:0_16:0_16:1/TG 12:0_16:0_18:1	1434	+	C_49_H_92_O_6_	794.7228 [M + NH_4_]^+^ 815.6531 [M + K]^+^ 799.6793 [M + Na]^+^	777.6 [M + H]-NH_3_ (15); 577.5 [M + H]-NH_3_-C_12_H_24_O_2_ (30); 549.4 [M + H]-NH_3_-C_14_H_28_O_2_ (80); 523.4 [M + H]-NH_3_-C_16_H_30_O_2_ (50); 521.5 [M + H]-NH_3_-C_16_H_32_O_2_ (100);	−0.42	II	E,V	S > C O > L
C069	TG 17:1_18:2_18:2	1434	+	C_56_H_98_O_6_	884.7704 [M + NH_4_]^+^ 905.6996 [M + K]^+^	867.7 [M + H]-NH_3_ (100); 849.5 [M + H]-NH_3_-H_2_O (15); 599.5 [M + H]-NH_3_-C_17_H_32_O_2_ (40); 587.4 [M + H]-NH_3_-C_18_H_32_O_2_ (50);	0.37	II	E,V	C > S L > O
C070	TG 16:0_16:1_16:1/…	1435	+	C_51_H_94_O_6_	820.7380 [M + NH_4_]^+^ 825.6947 [M + Na]^+^ 841.6684 [M + K]^+^	803.6 [M + H]-NH_3_ (15); 549.5 [M + H]-NH_3_-C_16_H_30_O_2_ (100); 547.5 [M + H]-NH_3_-C_16_H_32_O_2_ (60);	−0.97	II	E,V	S > C O > L
C071	TG 55:6	1436	+	C_58_H_100_O_6_	910.7855 [M + NH_4_]^+^	n.d.	−0.26	III	V	L > O
C072	TG 18:1_18:2_20:4/ TG 16:0_18:2_22:5	1436	+	C_59_H_100_O_6_	922.7852 [M + NH_4_]^+^ 943.715 [M + K]^+^	905.7 [M + H]-NH_3_ (60); 887.7 [M + H]-NH_3_-H_2_O (15); 649.5 [M + H]-NH_3_-C_16_H_32_O_2_ (15) 625.5 [M + H]-NH_3_-C_18_H_32_O_2_ (30) 623.6 [M + H]-NH_3_-C_18_H_34_O_2_ (25) 601.5 [M + H]-NH_3_-C_20_H_32_O_2_ (80) 577.5 [M + H]-NH_3_-C_18_H_332_O_2_ (10) 575.5 [M + H]-NH_3_-C_22_H_34_O_2_ (100)	−0.59	II	E,V	C > S L > O
C073	TG 58:8	1436	+	C_61_H_102_O_6_	948.8005 [M + NH_4_]^+^ 969.7299 [M + K]^+^	n.d.	−0.95	II	E,V	C > S L > O
C074	TG 16:1_16:1_18:1	1437	+	C_53_H_96_O_6_	846.7535 [M + NH_4_]^+^ 867.6837 [M + K]^+^	829.7 [M + H]-NH_3_ (20) 575.4 [M + H]-NH_3_-C_16_H_30_O_2_ (100) 547.5 [M + H]-NH_3_-C_18_H_34_O_2_ (45)	−1.12	II	E,V	S > C O > L
C075	TG 16:1_18:1_18:2	1440	+	C_55_H_98_O_6_	872.769 [M + NH_4_]^+^ 855.7436 [M + H]^+^	855.7 [M + H]-NH_3_ (50) 837.6 [M + H]-NH_3_-H_2_O (15); 601.5 [M + H]-NH_3_-C_16_H_30_O_2_ (95); 575.4 [M + H]-NH_3_-C_18_H_32_O_2_ (60) 573.4 [M + H]-NH_3_-C_18_H_34_O_2_ (100)	−1.26	II	E,V	C > S L > O
C076	TG 18:1_18:2_18:2	1441	+	C_57_H_100_O_6_	898.7841 [M + NH_4_]^+^ 881.7586 [M + H]^+^ 919.7147 [M + K]^+^	881.7 [M + H]-NH_3_ (100) 863.6 [M + H]-NH_3_-H_2_O (15); 601.5 [M + H]-NH_3_-C_18_H_32_O_2_ (70) 599.6 [M + H]-NH_3_-C_18_H_34_O_2_ (80)	−1.85	II	E,V	C > S L > O
C077	TG 15:0_16:0_16:1	1442	+	C_50_H_94_O_6_	808.7391 [M + NH_4_]^+^	791.6 [M + H]-NH_3_ (10) 549.6 [M + H]-NH_3_-C_15_H_30_O_2_ (100) 537.5 [M + H]-NH_3_-C_16_H_30_O_2_ (60) 535.4 [M + H]-NH_3_-C_16_H_32_O_2_ (90)	0.40		E,V	S > C O > L
C078	TG 15:0_16:0_18:2	1444	+	C_52_H_96_O_6_	834.7546 [M + NH_4_]^+^	575.4 [M + H]-NH_3_-C_15_H_30_O_2_ (90); 561.5 [M + H]-NH_3_-C_16_H_32_O_2_ (100); 537.5 [M + H]-NH_3_-C_18_H_32_O_2_ (80)	0.21	II	V	L > O
C079	TG 15:0_18:1_18:2/ others	1444	+	C_54_H_98_O_6_	860.7696 [M + NH_4_]^+^ 881.6997 [M + K]^+^	843.6 [M + H]-NH_3_ (50) 601.5 [M + H]-NH_3_-C_15_H_30_O_2_ (95) 587.5 [M + H]-NH_3_-C_16_H_32_O_2_ (65); 575.4 [M + H]-NH_3_-C_17_H_32_O_2_ (50); 563.5 [M + H]-NH_3_-C_18_H_32_O_2_ (100); 561.5 [M + H]-NH_3_-C_18_H_34_O_2_ (60);	−0.57		E,V	C > S L > O
C080	TG 57:7	1444	+	C_60_H_102_O_6_	936.8009 [M + NH_4_]^+^	n.d.	−0.52	III	E,V	C > S L > O
C081	TG 60:9	1444	+	C_63_H_104_O_6_	974.8164 [M + NH_4_]^+^	n.d.	−0.66	III	V	O > L
C082	TG 17:1_18:1_18:2	1445	+	C_56_H_100_O_6_	886.7857 [M + NH_4_]^+^ 891.7404 [M + Na]^+^ 907.7150 [M + K]^+^	869.7 [M + H]-NH_3_ (100); 851.7 [M + H]-NH_3_-H_2_O (15); 601.4 [M + H]-NH3-C_17_H_32_O_2_ (85); 589.6 [M + H]-NH3-C_18_H_32_O_2_ (90); 587.5 [M + H]-NH3-C_18_H_34_O_2_ (80)	−0.03	II	E,V	C > S L > O
C083	TG 58:7	1445	+	C_61_H_104_O_6_	950.8164 [M + NH_4_]^+^ 971.746 [M + K]^+^	n.d.	−0.68	III	E,V	C > S L > O
C084	TG 14:0_16:0_16:0	1446	+	C_49_H_94_O_6_	796.7367 [M + NH_4_]^+^	551.5 [M + H]-NH_3_-C_16_H_30_O_2_ (100); 523.4 [M + H]-NH_3_-C_14_H_28_O_2_ (40)	−2.67	II	E	S > C
C085	TG 18:2_18:2_19:1	1446	+	C_58_H_102_O_6_	912.8013 [M + NH_4_]^+^ 933.7308 [M + K]^+^	869.7 [M + H]-NH_3_ (100); 615.5 [M + H]-NH_3_-C_18_H_32_O_2_ (85); 599.6 [M + H]-NH_3_-C_19_H_36_O_2_ (60)	−0.09	II	E,V	C > S L > O
C086	TG 16:0_16:0_16:1/TG 14:0_16:0_18:1	1447	+	C_51_H_96_O_6_	822.7539 [M + NH_4_]^+^ 843.6841 [M + K]^+^	805.7 [M + H]-NH_3_ (20); 551.5 [M + H]-NH_3_-C_16_H_30_O_2_ (45); 549.6 [M + H]-NH_3_-C_16_H_32_O_2_ (100);	−0.66	II	E,V	S > C O > L
C087	TG 16:0_16:0_18:2 TG 16:0_16:1_18:1	1449	+	C_53_H_98_O_6_	848.7691 [M + NH_4_]^+^	577.4 [M + H]-NH_3_-C_16_H_30_O_2_ (80); 575.4 [M + H]-NH_3_-C_16_H_32_O_2_ (100); 549.5 [M + H]-NH_3_-C_18_H_34_O_2_ (90)	−1.18	II	E,V	S > C O > L
C088	TG 18:1_18:1_18:2	1452	+	C_57_H_102_O_6_	900.7994 [M + NH_4_]^+^	883.8 [M + H]-NH_3_ (30); 603.5 [M + H]-NH_3_-C_18_H_32_O_2_ (40); 601.5 [M + H]-NH_3_-C_18_H_34_O_2_ (100)	−2.24	II	E,V	C > S L > O
C089	TG 15:0_16:0_18:1	1454	+	C_52_H_98_O_6_	836.7700 [M + NH_4_]^+^ 857.7001 [M + K]^+^	819.7 [M + H]-NH_3_ (20); 577.5 [M + H]-NH_3_-C_15_H_30_O_2_ (100); 563.6 [M + H]-NH_3_-C_16_H_32_O_2_ (80); 537.4 [M + H]-NH_3_-C_18_H_34_O_2_ (90)	−0.10	II	E,V	S > C O > L
C090	TG 57:6	1454	+	C_60_H_104_O_6_	938.8167 [M + NH_4_]^+^	n.d.	−0.36	III	E,V	C > S L > O
C091	TG 17:1_18:1_18:1	1456	+	C_56_H_102_O_6_	888.8004 [M + NH_4_]^+^ 893.7565 [M + Na]^+^ 909.7307 [M + K]^+^	871.7 [M + H]-NH_3_ (20); 603.4 [M + H]-NH_3_-C_17_H_32_O_2_ (40); 589.6 [M + H]-NH3-C_18_H_34_O_2_ (100)	−1.13	II	E,V	C > S L > O
C092	TG 56:5	1456	+	C_59_H_104_O_6_	926.8153 [M + NH_4_]^+^ 947.7461 [M + K]^+^ 931.7720 [M + Na]^+^	n.d.	−1.90	III	E,V	S > C O > L
C093	TG 18:1_18:2_19:1	1457	+	C_58_H_104_O_6_	914.8167 [M + NH_4_]^+^ 935.7462 [M + K]^+^ 942.8487 [M + C_2_H_8_N]^+^	897.7 [M + H]-NH_3_ (60); 617.6 [M + H]-NH_3_-C_18_H_32_O_2_ (90); 615.5 [M + H]-NH_3_-C_18_H_34_O_2_ (100); 601.4 [M + H]-NH_3_-C_19_H_36_O_2_ (40)	−0.37	II	E,V	C > S L > O
C094	TG 18:1_18:1_22:4	1457	+	C_61_H_106_O_6_	952.8319 [M + NH_4_]^+^	935.7 [M + H]-NH_3_ (100); 917.7 [M + H]-NH_3_-H_2_O (15); 653.6 [M + H]-NH_3_-C_18_H_34_O_2_ (60); 603.5 [M + H]-NH_3_-C_22_H_36_O_2_ (50)	−0.83	II	V	O > L
C095	TG 60:7	1457	+	C_63_H_108_O_6_	978.8476 [M + NH_4_]^+^	n.d.	−0.76	III	E,V	C > S L > O
C096	TG 57:5	1458	+	C_60_H_106_O_6_	940.8322 [M + NH_4_]^+^	n.d.	−0.52	III	V	L > O
C097	TG 16:0_16:0_18:1	1460	+	C_53_H_100_O_6_	850.7846 [M + NH_4_]^+^	577.4 [M + H]-NH_3_-C_16_H_32_O_2_ (100); 551.5 [M + H]-NH_3_-C_18_H_34_O_2_ (45)	−1.36	II	E,V	S > C O > L
C098	TG 16:0_18:1_18:1	1460	+	C_55_H_102_O_6_	876.8001 [M + NH_4_]^+^	603.4 [M + H]-NH_3_-C_16_H_32_O_2_ (60); 577.5 [M + H]-NH_3_-C_18_H_34_O_2_ (100)	−1.49	II	E,V	S > C O > L
C099	TG 18:1_18:2_20:1	1465	+	C_59_H_106_O_6_	928.8317 [M + NH_4_]^+^	911.7 [M + H]-NH_3_ (50); 893.8 [M + H]-NH_3_-H_2_O (15); 631.5 [M + H]-NH_3_-C_18_H_32_O_2_ (40); 629.5 [M + H]-NH_3_-C_18_H_34_O_2_ (100); 601.5 [M + H]-NH_3_-C_20_H_38_O_2_ (60)	−1.08	II	V	L > O
C100	TG 16:0_16:0_17:0	1466	+	C_52_H_100_O_6_	838.7862 [M + NH_4_]^+^	565.5 [M + H]-NH_3_-C_16_H_32_O_2_ (100); 551.5 [M + H]-NH_3_-C_17_H_34_O_2_ (80)	0.57	II	V	L > O
C101	TG 18:1_18:1_19:1	1468	+	C_58_H_106_O_6_	916.8325 [M + NH_4_]^+^ 937.7621 [M + K]^+^; 921.788 [M + Na]^+^	899.8 [M + H]-NH_3_ (30) 881.7 [M + H]-NH_3_-H_2_O (15); 617.6 [M + H]-NH_3_-C_18_H_34_O_2_ (100); 603.6 [M + H]-NH_3_-C_19_H_36_O_2_ (50)	−0.20	II	E,V	C > S L > O
C102	TG 18:0_18:1_22:4	1468	+	C_61_H_108_O_6_	975.7774 [M + K]^+^ 954.8479 [M + NH_4_]^+^	937.8 [M + H]-NH_3_ (90) 919.6 [M + H]-NH_3_-H_2_O (30); 655.6 [M + H]-NH_3_-C_18_H_34_O_2_ (70); 653.5 [M + H]-NH_3_-C_18_H_36_O_2_ (55); 605.5 [M + H]-NH_3_-C_22_H_36_O_2_ (65)	−0.46	II	E,V	S > C O > L
C103	TG 57:4	1469	+	C_60_H_108_O_6_	942.8485 [M + NH_4_]^+^	n.d.	0.18	III	V	L > O
C104	TG 59:5	1470	+	C_62_H_110_O_6_	968.8638 [M + NH_4_]^+^	n.d.	−0.19	III	V	L > O
C105	TG 16:0_18:0_18:1	1473	+	C_55_H_104_O_6_	878.8162 [M + NH_4_]^+^ 899.7463 [M + K]^+^ 883.7727 [M + Na]^+^	605.4 [M + H]-NH_3_-C_16_H_32_O_2_ (100); 579.4 [M + H]-NH_3_-C_18_H_34_O_2_ (70); 577.5 [M + H]-NH_3_-C_18_H_36_O_2_ (90)	−0.96	II	E,V	S > C O > L
C106	TG 18:0_18:1_18:1/TG 16:0_18:1_20:1	1473	+	C_57_H_106_O_6_	904.8314 [M + NH_4_]^+^	605.4 [M + H]-NH_3_-C_18_H_34_O_2_ (100); 603.4 [M + H]-NH_3_-C_18_H_36_O_2_ (50)	−1.44	II	E,V	S > C O > L
C107	TG 18:1_18:1_20:1	1475	+	C_59_H_108_O_6_	930.8472 [M + NH_4_]^+^ 935.8038 [M + Na]^+^ 951.7773 [M + K]^+^	913.8 [M + H]-NH_3_ (20); 631.5 [M + H]-NH_3_-C_18_H_34_O_2_ (100); 603.5 [M + H]-NH_3_-C_20_H_38_O_2_ (65)	−1.24	II	V	L > O
C108	TG 18:0_18:1_22:3	1476	+	C_61_H_110_O_6_	956.8635 [M + NH_4_]^+^ 977.793 [M + K]^+^	939.8 [M + H]-NH_3_ (100); 921.6 [M + H]-NH_3_-H_2_O (30); 657.5 [M + H]-NH_3_-C_18_H_34_O_2_ (40); 655.6 [M + H]-NH_3_-C_18_H_36_O_2_ (65); 605.5 [M + H]-NH_3_-C_22_H_38_O_2_ (45)	−0.51	II	E,V	C > S L > O
C109	TG 18:2_18:2_24:1	1476	+	C_63_H_112_O_6_	982.8792 [M + NH_4_]^+^	695.8 [M + H]-NH_3_ (100); 947.8 [M + H]-NH_3_-H_2_O (20); 685.7 [M + H]-NH_3_-C_18_H_32_O_2_ (60); 599.4 [M + H]-NH_3_-C_24_H_46_O_2_ (50)	−0.45	II	E,V	C > S L > O
C110	TG 16:0_17:0_18:0/ others	1479	+	C_54_H_104_O_6_	866.8178 [M + NH_4_]^+^	607.5 [M + H]-NH_3_-C_15_H_30_O_2_ (30); 593.6 [M + H]-NH_3_-C_16_H_32_O_2_ (100); 579.6 [M + H]-NH_3_-C_17_H_34_O_2_ (80); 565.4 [M + H]-NH_3_-C_18_H_36_O_2_ (75); 551.5 [M + H]-NH_3_-C_19_H_38_O_2_ (30);	0.91	II	V	L > O
C111	TG 18:1_18:1_19:0	1479	+	C_58_H_108_O_6_	918.8481 [M + NH_4_]^+^ 923.8041 [M + Na]^+^ 939.7776 [M + K]^+^	619.6 [M + H]-NH_3_-C_18_H_34_O_2_ (100); 603.5 [M + H]-NH_3_-C_19_H_38_O_2_ (50)	−0.26	II	E,V	C > S L > O
C112	TG 18:2_18:2_23:0/TG 18:1_18:2_23:1	1481	+	C_62_H_112_O_6_	970.8798 [M + NH_4_]^+^	953.9 [M + H]-NH_3_ (100); 935.7 [M + H]-NH_3_-H_2_O (15); 673.6 [M + H]-NH_3_-C_18_H_32_O_2_ (90); 671.7 [M + H]-NH_3_-C_18_H_34_O_2_ (70); 601.6 [M + H]-NH_3_-C_23_H_44_O_2_ (50); 599.6 [M + H]-NH_3_-C_23_H_46_O_2_ (50)	0.18	II	E,V	C > S L > O
C113	TG 18:0_18:0_18:1/TG 16:0_18:1_20:0	1485	+	C_57_H_108_O_6_	906.8474 [M + NH_4_]^+^ 927.7779 [M + K]^+^ 911.8043 [M + Na]^+^	607.5 [M + H]-NH_3_-C_18_H_34_O_2_ (70); 605.5 [M + H]-NH_3_-C_18_H_36_O_2_ (100);	−1.05	II	E,V	S > C O > L
C114	TG 60:4	1485	+	C_63_H_114_O_6_	984.895 [M + NH_4_]^+^	n.d.	−0.29	III	E,V	C > S L > O
C115	TG 58:3	1486	+	C_61_H_112_O_6_	958.8791 [M + NH_4_]^+^ 979.8088 [M + K]^+^ 963.8349 [M + Na]^+^	n.d.	−0.56	III	E,V	C > S L > O
C116	TG 18:1_18:1_21:0/ TG 16:0_18:1_23:1/ TG 16:0_18:0_23:2	1493	+	C_60_H_112_O_6_	946.8797 [M + NH_4_]^+^ 967.8087 [M + K]^+^	673.7 [M + H]-NH_3_-C_16_H_32_O_2_ (90); 647.6 [M + H]-NH_3_-C_18_H_34_O_2_ (60); 645.6 [M + H]-NH_3_-C_18_H_36_O_2_ (100); 603.6 [M + H]-NH_3_-C_21_H_42_O_2_ (30); 577.5 [M + H]-NH_3_-C_23_H_44_O_2_ (30); 575.5 [M + H]-NH_3_-C_23_H_42_O_2_ (100);	0.08	II	V	L > O
C117	TG 18:1_18:2_23:0	1493	+	C_62_H_114_O_6_	972.8958 [M + NH_4_]^+^ 993.8246 [M + K]^+^	955.9 [M + H]-NH_3_ (30); 937.7 [M + H]-NH_3_-H_2_O (20); 675.6 [M + H]-NH_3_-C_18_H_32_O_2_ (80); 673.6 [M + H]-NH_3_-C_18_H_34_O_2_ (90); 601.4 [M + H]-NH_3_-C_23_H_46_O_2_ (100);	0.34	II	V	L > O
C118	TG 18:2_18:2_25:0	1494	+	C_64_H_116_O_6_	998.9106 [M + NH_4_]^+^	981.7 [M + H]-NH_3_ (50); 963.7 [M + H]-NH_3_-H_2_O (20); 701.7 [M + H]-NH_3_-C_18_H_32_O_2_ (95); 599.5 [M + H]-NH_3_-C_25_H_50_O_2_ (100);	−0.34	II	V	L > O
C119	TG 16:0_18:1_24:1	1498	+	C_61_H_114_O_6_	960.8948 [M + NH_4_]^+^ 981.8245 [M + K]^+^ 965.8508 [M + Na]^+^	687.6 [M + H]-NH_3_-C_16_H_32_O_2_ (90); 661.6 [M + H]-NH_3_-C_18_H_34_O_2_ (100); 577.6 [M + H]-NH_3_-C_24_H_46_O_2_ (75);	−0.51	II	V	L > O
C120	TG 18:1_18:1_24:1	1498	+	C_63_H_116_O_6_	986.9104 [M + NH_4_]^+^	969.8 [M + H]-NH_3_ (15); 687.7 [M + H]-NH_3_-C_18_H_34_O_2_ (100); 603.5 [M + H]-NH_3_-C_24_H_46_O_2_ (60);	−0.55	II	E,V	C > S L > O
C121	TG 18:1_18:1_23:0	1505	+	C_62_H_116_O_6_	974.9109 [M + NH_4_]^+^	675.6 [M + H]-NH_3_-C_18_H_34_O_2_ (100); 603.6 [M + H]-NH_3_-C_23_H_46_O_2_ (40);	−0.03	II	V	L > O
C122	TG 16:0_18:1_26:1	1512	+	C_63_H_118_O_6_	988.9262 [M + NH_4_]^+^	715.6 [M + H]-NH_3_-C_16_H_32_O_2_ (75); 689.5 [M + H]-NH_3_-C_18_H_34_O_2_ (100); 577.5 [M + H]-NH_3_-C_26_H_50_O_2_ (60);	−0.39	II	V	L > O

C (compound code); rt (retention time); P (polarity); MF (molecular formula); MS (exact mass); error (ppm); ID (level of identification); T (tissue); DG (diglyceride); TG (triglyceride); L (liver); E (epididymal); V (visceral); n.d. (non determined). Exact mass reported in bold was used for the MS/MS experiments.

## Data Availability

The raw data from metabolomics and lipidomics are available at the metabolights repository MTBLS5983 (www.ebi.ac.uk/metabolights/MTBLS5983, accessed on 18 October 2022).

## Supplementary Materials

The following supporting information can be downloaded at: https://www.mdpi.com/article/10.3390/antiox11112165/s1, S1. Standards and reagents; S2. *Sofrito* bioactive compounds analysis; S3. Table S1. List of primers used for the RT-PCR assays in liver and adipose tissue; Table S2. Body and organs weight, food intake and serum biochemical determinations; Table S3. Characterization of carotenoids and phenolic compound contents in tomato *sofrito*.
