# Supplementary material for: Integrated Metabolomics, Lipidomics, and Genomics Reveal the Presence of a New Biomarker, Butanediol Glucuronide, Associated with the Activation of Liver Ketogenesis and Lipid Oxidation by Tomato-Based Sofrito in Obese Rats
Source: Antioxidants (Basel). 2022 Oct 31;11(11):2165. doi: 10.3390/antiox11112165 (PMC9686708; doi:10.3390/antiox11112165)
Supplement: Supplementary file 1 [file antioxidants-11-02165-s001.zip › antioxidants-2010091-supplementary.pdf]

Supplemental Material S1

1. Standards and reagents

1.1 Standards and reagents for sofrito *analysis*

Acetonitrile, ethanol, methanol, formic acid, and acetic acid were purchased from AppliChem, Panreac Quimica SA (Barcelona, Spain). Hexane, methyl tert-butyl ether (MTBE), all-E-α-carotene, all-E-β-carotene, all-E-lutein, all-E-lycopene caffeic acid, p-coumaric acid, chlorogenic acid, ferulic acid, isolariciresinol, larisiresinol, luteolin, naringenin, oleuropein, pinoresinol, protocatchuic acid, quercetin, rutin, and secoisolariciresinol were purchased from Sigma-Aldrich (St. Louis, MO, USA). Narigenin-7-O-glucoside, hydroxytyrosol, and ethyl gallate were acquired from Extrasynthese (Genay, France), vanillic acid and apigenin from Fluka (St. Louis, MO, USA), and verbascoside from HWI Analytic. Ultrapure water was obtained using a Milli-Q purification system (Millipore, Bedford, MA, USA).

1.2 Standards and reagents for metabolomics and lipidomics approach

LC-MS grade acetonitrile, methanol, formic acid and isopropanol, and ammonium formate were obtained from Sigma-Aldrich (St. Louis, MO, USA). . Ultrapure water was obtained using a Milli-Q purification system (Millipore, Bedford, MA, USA). Bile acids such as cholic acid-D4 (CA-D4), litocholic acid-D4 (LCA-D4), deoxycholic acid-D4 (DCA-D4), chenodeoxycholic acid-D4 (CDCA-D4), glicocholic acid-D4 (GCA-D4), glicolitocholic acid-D4 (GLCA-D4), glicochenodeoxycholic acid-D4 (GCDCA-D4), glicoursodeoxycholic acid-D4 (GUDCA-D4) were bought from CDN isotopes / Spectra2000 and ursodeoxycholic acid-D4 (UDCA-D4), taurocholic acid-D5 (TCA-D5), taurolitocholic acid-D5 (TLCA-D5), taurochenodeoxycholic acid-D5 (TCDCA-D5) were

obtained from TRC Canada / Spectra2000. L-tyrosine-D5, hippuric acid-D5 and cinnamic acid

1.3 Standard solutions for MS-based untargeted approach

IS-1 (L-tyr-D5; HA-D5; CiA-D5; GCA-D4 and CA-D4) was prepared for metabolomics approach, stock solution of 10 ppm in pure methanol.

IS-2 (CA-D4; LCA-D4; DCA-D4; CDCA-D4; UDCA-D4; GCA-D4; GLCA-D4; GCDCA-D4; GUDCA-D4; TCA-D5; TLCA-D5; TCDCA-D5) was prepared for lipidomics approach, stock solution of 10 ppm in pure methanol.

Supplemental Material S2

1. Sofrito Bioactive compounds analysis

1.1 Carotenoids analysis

For carotenoid extraction, *sofrito* (0.5 g) was weight and homogenized with ethanol:n-henaxe (4:3, v/v), after which it was sonicated for 10 min on ice and then centrifuged at 2486 g, for 20 min at 4 ºC. The apolar phase was separated and the extraction was repeated until colorless. The supernatants were combined and evaporated under nitrogen flow until dry and the residue was suspended with MTBE and filtered using a 0.22 µm PTFE filter and stored at -80 ºC until analysis.

Carotenoid analysis was performed using a HPLC system equipped with a quaternary pump and an autosampler (HP1100 HPLC system, Hewllet-Packard, Waldbronn, Germany) coupled to a DAD (G1315B). Separation was carried out on a C_{30} column (250 x 4.6 mm, 5 µm, YMC, Walters Co. Milford, USA). The chromatography condition were based on the procedure reported by (Vallverd-Queralt et al., 2013). Identification of carotenoids was confirmed by retention time chromatography with standards, UV/VIS absorption spectrum, spectral fine structure and peak cis intensity compared to standards and literature (Rinaldi de Alvarenga et al., 2019). To confirm the identification, a HPLC-APCI-QqQ-MS/MS (QTRAP4000, Sciex, Foster City, CA, USA) equipped in positive-ion mode was used (Hrvolov et al., 2016). Quantification was performed using external calibration curves for all-*E*-α-carotene, all-*E*-β-carotene and all-*E*-lycopene. The Z-isomers were quantified by calibration curves corresponding to the *E*-form and when there is no standard available, expressed as β-carotene equivalent. The results were expressed as µg/g of sofrito.

1.2 Polyphenols analysis

For polyphenols extraction, *sofrito* (0.5 g) was homogenized with ethanol:water (80:20, v/v), then sonicated, centrifuged and extracted applying the same conditions. The residue was reconstituted up to 2 mL of ultrapure water with 0.1% of formic acid, filtered through a 0.22 µm PTFE filter storage at -80 ºC (Vallverdú-Queralt, de Alvarenga, Estruch, & Lamuela-Raventos, 2013).

Phenolic compounds were identified and quantified by UPLC-ESI-QqQ-MS/MS using the conditions of a validated method by Di Lecce et al. (2013) for tomato polyphenols and a method described by Capriotti et al. (2014) for olive oil polyphenols. An UHPLC Acquity system equipped with a binary pump and an autosampler from Waters (Milford, MA, USA). A BEH C_{18} column (50 mm × 2.1 mm) i.d., 1.7µm (Waters, Milford, MA, United States) was used. An API3000 triple quadrupole mass spectrometer (ABSciex, Concord, Ontario, Canada) equipped with Turbo Ionspray source in negative-ion mode was used for the MS/MS data analysis. Quantification of polyphenols was performed using multiple reactions monitoring mode tracking the transition of parent ion and product ions specific for each compound and energy conditions were described in Rinaldi de Alvarenga et al. (2019). The quantification was carried out by the internal standard method using ethyl gallate. The polyphenols were quantified related to their corresponding standard or with a compound corresponding at the same class. The results were expressed as µg/g of sofrito.

Supplemental Material S3

Table S1. List of the primers used for the RT-PCR assays in liver and adipose tissue.

**Gene**		**Sequence (5' – 3')**
Atgl	For	TGTGGCCTCATTCCTCCTAC
	Rev	AGCCCTGTTTGCACATCTCT
β-actin	For	AAGTCCCTCACCCTCCCAAAG
	Rev	AAGCAATGCTGTCACCTTCCC
Cgi-58	For	CTACCTGGTGTCCCACGTCT
	Rev	CAAGACCTCCTCCAAAACCA
Cidea	For	TGATATCCGCTGCACAAGC
	Rev	CACCTGGGCAGCATAGGA
Cpt1a	For	ACAATGGGACATTCCAGGAG
	Rev	AAAGACTGGCGCTGCTCA
Dgat1	For	AATGCTGCGGAAAAACTACG
	Rev	TTGCTGGTAACAGTGCTTGC
Dgat2	For	AATCTGTGGTGCCGCCAG
	Rev	TCCCTGCAGACACAGCTTTG
G6Pase	For	GACCTCAGGAACGCCTTCTATG
	Rev	ATTGATGCCCACAGTCTCTTGA
Hmgcs2	For	TGAACGGTGAATAGACACAGCG
	Rev	GTGGTGCTCACTGCTTCAGG
Hsl	For	TCACGCTACATAAAGGCTGCT
	Rev	CCACCCGTAAAGAGGGAACT
Leptin	For	CCAGGATCAATGACATTTCACA
	Rev	AATGAAGTCCAAACCGGTGA
Lpl	For	TATGGCACAGTGGCTGAAAG
	Rev	CTGACCAGCGGAAGTAGGAG
Pepck	For	GTCACCATCACTTCCTGGAAGA
	Rev	GGTGCAGAATCGCGAGTTG
Pgc1apha	For	AAGATGCCTCCTGT GACT
	Rev	GATGACCGAAGTGCTTGT
Pkl	For	GACCCGAAGTTCCAGACAAGG
	Rev	ATGAGCCCGTCGTCAATGTAG
Prdm16	For	CGGATGTTCCCCAACAAAT
	Rev	ACGCTCTTCTGTGTGGACAA
S18	For	CCACAGGAGGCCTACACG
	Rev	CTGAAACTTCTCGGGGATCA
Ucp1	For	GCCTGCCTAGCAGACATCAT
	Rev	TGGCCTTCACCTTGGATCT

Table S2. Body and organs weight, food intake and serum biochemical determinations.

	LC	LS	OC	OS
Body weight (g)	383.6 ± 28.7	378.4 ± 20.7	518.3* ± 49.6	517.3^{+} ± 46.1
Food intake (g/day/rat)	20.00 ± 3.73	23.61 ± 4.43	28.64* ± 5.32	34.44^{+} ± 3.39
Caloric intake	61.99 ± 11.50	73.71 ± 13.80	88.79* ± 16.50	$107.55^{+\#}$ ± 10.57
ITT (glucose mg/dL at 120 min)	53.53 ± 1.10	57.89 ± 5.37	102.97 ± 6.06*	$84.55 \pm 4.73^{+\#}$
Organ weight (g/100g body weight)				
Liver	3.78 ± 0.43	3.46 ± 0.14	5.11* ± 0.86	5.44^{+} ± 0.92
VAT	0.78 ± 0.07	0.91 ± 0.07	1.58* ± 0.23	1.35^{+} ± 0.19
EAT	0.75 ± 0.14	0.77 ± 0.26	1.94* ± 0.21	1.89^{+} ± 0.24
Serum biochemical				
Glucose (mmol/L)	7.1 ± 026	7.63 ± 0.20	8.43* ± 0.81	9.4^{+} ± 2.26
Triglycerides (mmol/L)	1.50 ± 0.31	1.33 ± 0.25	5.68* ± 1.21	4.76^{+} ± 0.62
Cholesterol (mmol/L)	3.50 ± 0.20	3.52 ± 0.23	6.09* ± 0.95	7.07^{+} ± 0.78

Mean ± SD

*$p < 0.05$ versus LC; $^{+}p < 0.05$ versus LS; $^{\#}p < 0.05$ versus OC.

LC, lean rats fed a control diet; LS, lean rats fed a sofrito-supplemented diet; OC, obese rats fed a control diet; OS, obese rats fed a sofrito-supplemented diet; VAT, visceral perirenal + retroperitoneal adipose tissue; EAT, epididymal adipose tissue. Results previously published by Rodriguez-Rodriguez et al. (2017).

Table S3. Characterization of carotenoids and phenolic compounds content in tomato *sofrito* used in the study in µg/g of fresh weight. *Sofrito* was composed by tomato (50%) (pulp and concentrated), onion (37%), extra virgin olive oil (12%), and salt (Rodriguez-Rodriguez et al. 2017).

Carotenoids	
all-*E*-ζ-carotene	3.6 ± 0.3
all-*E*-α-carotene	4.0 ± 0.2
all-*E*-β-carotene	9.1 ± 0.2
13-*Z*-β-carotene	3.31 ± 0.05
15-*Z*-lycopene	7.3 ± 0.5
13-*Z*-lycopene	33 ± 3
9-*Z*-lycopene	34 ± 1
all-*E*-licopene	142 ± 3
5-*Z*-lycopene	28.6 ± 0.2
Polyphenols	
3,4-DHPEA-EDA	0.052 ± 0.002
caffeic acid	0.74 ± 0.02
caffeic acid hexoside I	1.77 ± 0.02
chlorogenic acid	3.6 ± 0.3
coumaric acid hexoside I	0.51 ± 0.01
DHOA	0.046 ± 0.001
dicaffeoylquinic acid	0.20 ± 0.01
dihydroxytyrosol	0.105 ± 0.001
eneloic acid	0.074 ± 0.001
ferulic acid	0.51 ± 0.04
ferulic acid hexoside	2.16 ± 0.04
HCM-EA	0.34 ± 0.01
HDCM-OA	1.1 ± 0.1
HOA	0.26 ± 0.01
hydroxyelenoic acid	0.26 ± 0.03
hydroxytyrosol	1.00 ± 0.06
lariciresinol	0.32 ± 0.03
luteolin	1.5 ± 0.2
naringenin	9.6 ± 0.9
naringenin-7-glucoside	0.87 ± 0.01
oleuropein	0.063 ± 0.002
p-coumaric acid	0.27 ± 0.02
pinoresinol	1.1 ± 0.1
protocatechuic acid	0.28 ± 0.02
rutin	4.5 ± 0.7
vanillic acid	0.39 ± 0.02

Mean ± SD

3,4-DHPEA-EDA, decarboxyl methyl oleuropein aglycone; DHOA, dihydroxyoleuropein aglycone; HCM-EA, hydroxycarboxymethyl elenoic acid; HDCM-OA, hydroxydecarboxymethyl oleuropein aglycone; HOA, hydroxyoleuropein aglycone.
